# Long-term beneficial effect of faecal microbiota transplantation on colonisation of multidrug-resistant bacteria and resistome abundance in patients with recurrent *Clostridioides difficile* infection

**DOI:** 10.1186/s13073-024-01306-7

**Published:** 2024-02-28

**Authors:** Sam Nooij, Karuna E. W. Vendrik, Romy D. Zwittink, Quinten R. Ducarmon, Josbert J. Keller, Ed J. Kuijper, Elisabeth M. Terveer, on behalf of the Netherlands Donor Feces Bank study group

**Affiliations:** 1 Netherlands Donor Feces Bank, Leiden University Center of Infectious Diseases (LUCID) Medical Microbiology and Infection Prevention, Leiden University Medical Center, PO Box 9600, Postzone E4-P, Leiden, 2300RC Netherlands; 2 Center for Microbiome Analyses and Therapeutics, LUCID Research, Leiden University Medical Center, Leiden, Netherlands; 3 Department of Gastroenterology, Haaglanden Medical Center, The Hague, Netherlands; 4 Present address: Centre for Infectious Disease Control, Netherlands Institute for Public Health and the Environment, Bilthoven, The Netherlands

**Keywords:** C. diff, Antibiotic resistance, MDRO, Faecal microbiota transplantation

## Abstract

**Background:**

Multidrug-resistant (MDR) bacteria are a growing global threat, especially in healthcare facilities. Faecal microbiota transplantation (FMT) is an effective prevention strategy for recurrences of *Clostridioides difficile* infections and can also be useful for other microbiota-related diseases.

**Methods:**

We study the effect of FMT in patients with multiple recurrent *C. difficile* infections on colonisation with MDR bacteria and antibiotic resistance genes (ARG) on the short (3 weeks) and long term (1–3 years), combining culture methods and faecal metagenomics.

**Results:**

Based on MDR culture (*n* = 87 patients), we notice a decrease of 11.5% in the colonisation rate of MDR bacteria after FMT (20/87 before FMT = 23%, 10/87 3 weeks after FMT). Metagenomic sequencing of patient stool samples (*n* = 63) shows a reduction in relative abundances of ARGs in faeces, while the number of different resistance genes in patients remained higher compared to stools of their corresponding healthy donors (*n* = 11). Furthermore, plasmid predictions in metagenomic data indicate that patients harboured increased levels of resistance plasmids, which appear unaffected by FMT. In the long term (*n* = 22 patients), the recipients’ resistomes are still donor-like, suggesting the effect of FMT may last for years.

**Conclusions:**

Taken together, we hypothesise that FMT restores the gut microbiota to a composition that is closer to the composition of healthy donors, and potential pathogens are either lost or decreased to very low abundances. This process, however, does not end in the days following FMT. It may take months for the gut microbiome to re-establish a balanced state. Even though a reservoir of resistance genes remains, a notable part of which on plasmids, FMT decreases the total load of resistance genes.

**Supplementary Information:**

The online version contains supplementary material available at 10.1186/s13073-024-01306-7.

## Background

The discovery of antibiotics altered the natural course of infectious diseases and saved millions of lives. Antibiotics might be the most significant development in modern medicine, but there are important trade-offs to their use. Antibiotic-resistant bacteria have emerged that are unaffected by standard therapies, which threatens effective prevention and treatment of infections. Antibiotic resistance is now considered a major threat to public health [1, 2]. Besides, broad spectrum antibiotic therapy disrupts the human microbiota, paradoxically resulting in an increased susceptibility to infections, for example by *Clostridioides difficile* [3–5].

*C. difficile* can asymptomatically reside in the gut but thrives in an antibiotic-affected microbiota. *C. difficile* causes an infection (CDI) varying from self-limiting and mild diarrhoea to life-threatening pseudomembranous colitis [6]. The disruption of the gut microbiota is essential in maintaining the recurrent nature of CDI, which is supported by the observation that replenishing the gut microbiota by faecal microbiota transplantation (FMT) results in prompt resolution of CDI recurrence (rCDI) [7, 8]. It is thought that FMT restores the gut microbiota diversity after antibiotic treatment, thus preventing outgrowth of *C. difficile* spores [9], and possibly decreasing the risk of other infections as well. FMT has been mentioned in treatment guidelines for rCDI for years [10–12], and rCDI is currently the only disease that is routinely treated with FMT.

A gut microbiota disrupted by antibiotics is also more susceptible to colonisation with multidrug-resistant (MDR) bacteria [13], which in turn increases the risk of infection in critically ill patients [14]. A prominent and problematic group of MDR bacteria are extended-spectrum beta-lactamase-producing (ESBL) Enterobacterales. Most infections with ESBL-producing Enterobacterales have high morbidity and mortality and are preceded by intestinal colonisation [15–17]. Hence, the prevention and eradication of ESBL-producing Enterobacterales from the intestinal tract is of global interest. Spontaneous decolonisation depends on comorbidities and type of species [18, 19], and innovative strategies to promote decolonisation of MDR bacteria are desired. So far, there is no recommended decolonisation method [20]. However, Millan et al. found that FMT in patients with rCDI decreased the number and diversity of antimicrobial resistance genes in their faeces [21]. This observation was followed by various case reports of patients colonised with ESBL-producing Enterobacterales who were successfully treated with FMT [22–31]. One underpowered randomised controlled trial (RCT) has been conducted (*n* = 39 patients) to assess decolonisation of MDR Enterobacterales by treatment with oral non-absorbable antibiotics and FMT [32]. No statistically significant advantage of FMT was found, although colonisation rates were slightly lower in FMT-treated patients compared to untreated control patients. Subsequently, questions were raised about the efficacy of FMT against MDR bacteria and experiments were suggested to further assess this [33]. Interestingly, data from another RCT using FMT for the decolonisation of MDR bacteria in renal transplant patients indicated that FMT-treated patients had longer time to recurrent infections than patients that did not receive FMT [34]. This underscores the need for longer-term sampling in similar FMT studies.

To further explore the effects of FMT in rCDI patients on antibiotic resistance of the gut microbiota, we assess colonisation with MDR bacteria with both culture and faecal metagenomics. We pay special attention to the resistome, defined as the collection of all antibiotic resistance genes (ARG) present. Additionally, we study the long-term effects on the microbiota up to 3 years after FMT in a subset of patients.

## Methods

### Study design

In this cohort study, we use stool samples of rCDI patients treated in 34 different healthcare centres across the Netherlands with FMT provided by the Netherlands Donor Feces Bank (NDFB, Leiden, the Netherlands) to assess the presence of MDR bacteria and the resistome. The NDFB uses standardised procedures for the collection, screening, preparation and storage of donor faecal suspensions, and treatment and follow-up of rCDI patients as described previously [35, 36]. In short, patients were first treated with antibiotics against *C. difficile* for at least 4 days until 24 h before FMT. The day before FMT, patients received a bowel lavage with macrogol solution [8]. Pre-FMT samples were collected during or shortly after antibiotic treatment and before bowel lavage. Approximately 3 weeks after FMT, a short-term post-FMT sample was requested. Pre- and short-term post-FMT stool samples of rCDI patients and their corresponding donors were collected between May 2016 and March 2021. Additionally, in February 2021 we approached FMT-treated patients of the cohort with informed consent to contact them for later research purposes (*n* = 53) for updated clinical information and requested a long-term follow-up (LTFU) stool sample. Clinical data, including recurrence of CDI after FMT, were recorded for further investigation. Stool samples were stored at − 80 °C until DNA extraction for metagenomics sequencing or stored in an end concentration of 10% glycerol until MDR culture testing.

### Definition of multidrug-resistant bacteria

Definitions and testing methods were used as described previously [37]. MDR bacteria were defined according to the definitions of the Dutch Working Group on Infection Prevention [38]. This includes ESBL-producing Enterobacterales; Enterobacterales and *Acinetobacter* spp. that are resistant to both fluoroquinolones and an aminoglycoside or produce carbapenemases; *Pseudomonas aeruginosa* that produces carbapenemase or is resistant to at least three of the following antibiotic classes or agents: fluoroquinolones, aminoglycosides, ceftazidime or piperacillin, and carbapenems; both penicillin and vancomycin-resistant *Enterococcus faecium* (VRE); or methicillin-resistant *Staphylococcus aureus* (MRSA).

### Culture and antimicrobial susceptibility testing of multidrug-resistant bacteria

To identify MDR bacteria and calculate the prevalence among FMT donors and recipients, stool samples were selectively cultured as described previously [37]. Briefly, an inoculating loop was used to scrape 10 µL faeces from frozen faeces aliquots (containing 10% glycerol). The faeces was enriched in 15 mL of tryptic soy broth and incubated for 18 h at 35 °C prior to plating on ChromID ESBL, ChromID OXA-48 agar, MacConkey tobramycin (8 mg/L) plus ciprofloxacin (0.5 mg/L) agar, and VRE agar (bioMérieux, Marcy l’Etoile, France). For MRSA detection, a separate brain heart infusion enrichment broth was used which was supplemented with 2.5 sodium chloride and 10 mg/L colistin sulphate and inoculation on MRSA-ID agar plate. All suspected MDR colonies were identified as bacterial species by matrix-assisted laser desorption ionisation-time of flight mass spectrometry (MALDI-TOF) Biotyper (Bruker Daltonik; Bremen, Germany). Antibiotic susceptibility was evaluated by VITEK2 (Card N199, bioMérieux) using the European Committee of Antimicrobial Susceptibility Testing (EUCAST) breakpoints version 11.0 [39]. ESBL production was confirmed using the double disk method. Isolates with a meropenem minimum inhibitory concentration > 0.25 mg/L (ETEST, bioMérieux) were investigated for carbapenemase production with a carbapenem inactivation method (CIM) test and an in-house multiplex PCR to detect *KPC*, *VIM*, *NDM*, *OXA-48* and *IMP* genes. VRE were confirmed by an in-house PCR targeting the *vanA* and *vanB* genes, and MRSA with the BD MAX assay targeting the *MREJ, mecA/mecC* and *Nuc* genes (BD, New Jersey, USA). Six known MDR bacteria-positive and seven MDR bacteria-negative defrosted faeces aliquots (also stored in 10% glycerol) of the NDFB donor screening served as positive and negative controls. Samples were called MDR culture positive if at least one MDR bacterium was cultured on selective media.

### Whole-genome sequencing of multidrug-resistant isolates

**Fig. 1:**
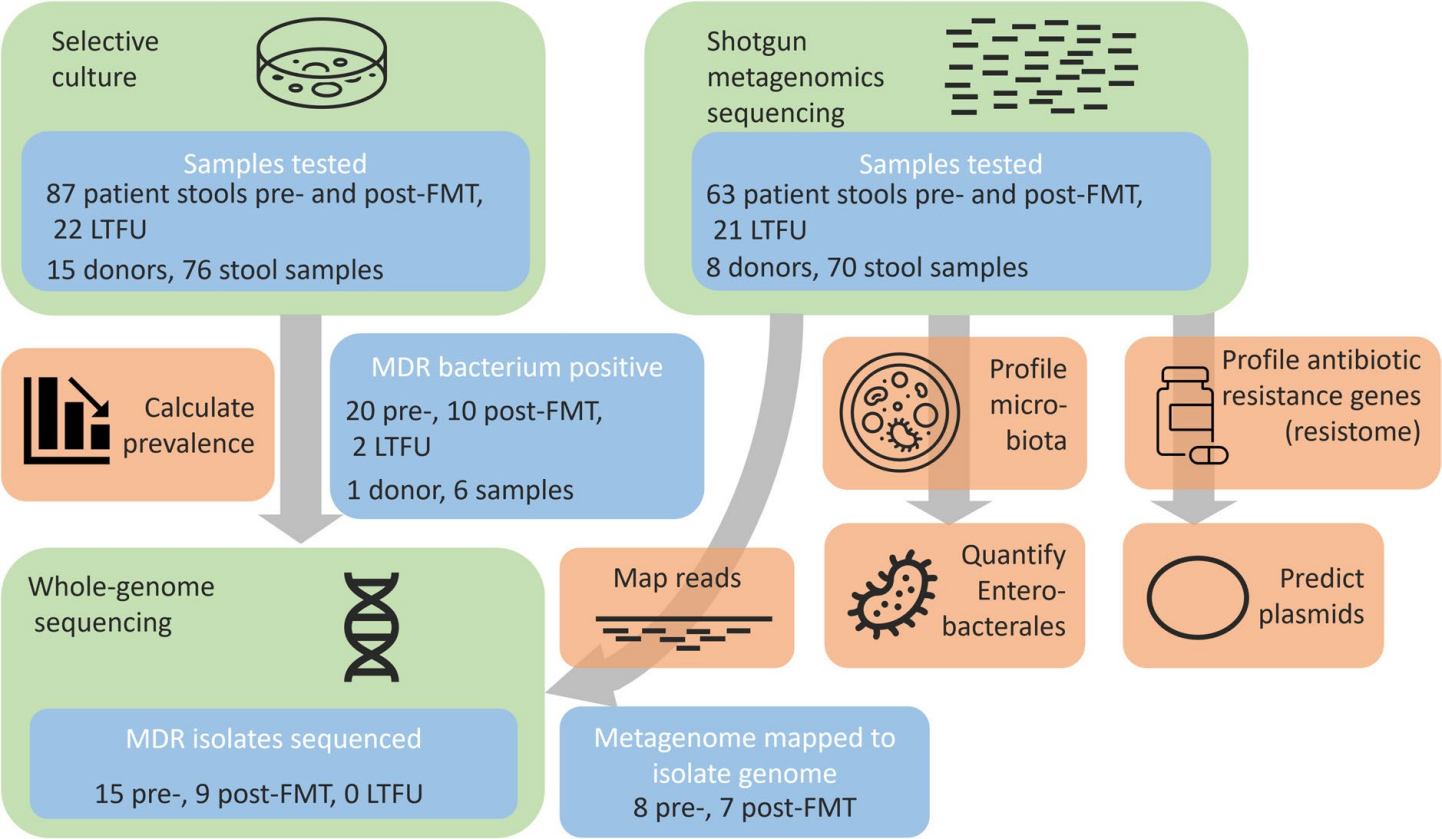
Schematic representation of the study setup. Data sources are shown in blue, data generating (wet lab) techniques in green and major analysis (dry lab) methods in orange boxes. Multiple recurrent *Clostridioides difficile* infected patients were treated with faecal microbiota transplantation in 34 different centres across the Netherlands and samples were requested for research. Only patients are included in the analyses if we received both a pre- and post-FMT sample

To assess the antibiotic resistance genotype of MDR isolates and persistence after FMT, cultured MDR bacteria were subjected to whole-genome sequencing (WGS; Fig. 1). DNA was isolated using the QIAsymphony DSP Virus/Pathogen Midi Kit (Qiagen, Hilden, Germany) and sent to GenomeScan B.V. (Leiden, Netherlands) to sequence on the Illumina NovaSeq6000 platform (Illumina, Inc., San Diego, California, USA) generating 150 bp paired-end reads (reads per bacterial isolate: 780 k [258 k-1.64 M] (median [range])). Samples were sent in two batches, of which the second failed. We decided to continue with the available data, which includes WGS for 24 out of 32 isolates (15 / 20 from pre-FMT stools, 9 / 10 from short-term post-FMT and 0 / 2 long-term follow-up). The raw sequencing reads were cleared of human-derived reads by mapping to the GRCh38 genome [40] using bowtie2 (version 2.4.2, option ‘–very-sensitive-local’) [41] and samtools (version 1.11) [42] before adapter and low-complexity read removal and quality-trimming using fastp (version 0.20.1, parameters ‘–cut_right –cut_window_size 4 –cut_mean_quality 20 -l 50 –detect_adapter_for_pe -y’) [43]. High-quality reads were assembled using SPAdes (version 3.15.2, option ‘–isolate’) [44]. All scaffolds were screened for antibiotic resistance genes using ABRicate (version 0.8.13, https://github.com/tseemann/abricate) with both the CARD (from 25 March 2021) [45] and ResFinder (from 25 March 2021) [46] databases, only retaining hits of full-length genes (100% coverage) with at least 97% identity. These cut-offs were used to keep the method consistent with and comparable to the resistome analyses (see below). Furthermore, assembled genomes were taxonomically classified using GTDB-Tk (version 2.1.0) [47]. These classifications were used to verify or further specify classifications made by MALDI-TOF Biotyper as described above and are used as species identification for sequenced isolates. Sequence data have been deposited in the European Nucleotide Archive (ENA) under project number PRJEB64622 (https://www.ebi.ac.uk/ena/browser/view/PRJEB64622) [48].

### Shotgun metagenomic sequencing

In total, 63 sets of donor-patient FMT triads were sequenced using shotgun metagenomics. Samples collected before 2021 were prepared for sequencing as previously described [49]. This resulted in metagenomes of 49 patients pre- and short-term post-FMT and 56 donor samples of 8 donors that have been deposited in the ENA under project number PRJEB44737 (https://www.ebi.ac.uk/ena/browser/view/PRJEB44737) [50]. An additional 21 sets (tetrads) of patient pre-, short-term post-FMT and now including long-term post-FMT samples, of which 7 were sequenced earlier, as well as 14 donor samples from 8 donors were sequenced at GenomeScan B.V. (Leiden, Netherlands) using the Illumina NovaSeq6000 platform generating a median of 42.6 M 150 bp paired-end reads per sample. Raw reads, excluding human-derived reads (see below), have been deposited in the ENA under project number PRJEB64621 (https://www.ebi.ac.uk/ena/browser/view/PRJEB64621) [51]. DNA was extracted from 100 mg of unprocessed patient and donor faeces using the Quick-DNA Fecal/Soil Microbe Miniprep Kit (ZymoResearch, Irvine, California, USA), with bead beating step on a Precellys 24 tissue homogeniser (Bertin Technologies, Montigny-le-Bretonneux, France) at 5.5 m/s for three times 1 min with short intervals, as described previously [52]. Libraries were constructed using the NEBNext Ultra II FS DNA kit and NEBNext Ultra II Ligation kit (New England Biolabs, Ipswich, Massachusetts, USA), producing DNA fragments of approximately 500–700 bp. Besides, control samples were included to verify successful DNA isolation and sequencing. These include blank (water) controls, and ZymoBiomics Community Standard (ZymoResearch). Negative controls returned no sequencing reads, while positive controls contained reads of all species present in the communities.

### Metagenomic pre-processing

Human-derived reads were removed from raw metagenomic reads by mapping reads to the human reference genome (GRCh38, NCBI accession ID GCF_000001405.26) using bowtie2 (version 2.4.2, option ‘–very-sensitive-local’) and samtools (version 1.11). Remaining non-human reads were then processed by fastp (version 0.20.1) to trim low-quality 3’-ends (parameters: ‘–cut_right –cut_window_size 4 –cut_mean_quality 20’), remove low-complexity sequences (parameter: ‘-y’), remove remaining adapter sequences (parameter: ‘–detect_adapter_for_pe’) and remove reads shorter than 50 bases (parameter: ‘-l 50’). The resulting high-quality metagenomic reads were used in read-based taxonomic profiling and assembly-based ARG profiling.

### Quantification of multidrug-resistant isolates in metagenomes

To identify and quantify whole-genome sequenced MDR bacteria in metagenomes, we mapped metagenomic reads derived from the same stool sample to the respective assembled genome using BWA-MEM (version 0.7.17) [53]. Mapped reads were counted and coverage was quantified using samtools coverage (version 1.10). Coverage was calculated as both depth of coverage (mean number of times each position is covered, normalised by the total number of metagenomic reads) and breadth of coverage (percentage of genome covered by at least one read). In all cases, presence of the MDR strain was confirmed by coverage of scaffolds containing ARGs related to the MDR phenotype. Furthermore, presence of antibiotic resistance genes detected in the whole-genome sequence data of each cultured isolate was manually compared against the resistome data derived from the same stool sample to assess sensitivity of culture and metagenomics.

### Taxonomic profiling

Taxonomic microbiota profiles were determined using MetaPhlAn (version 4.0.3) [54], which maps reads to its custom marker database. Resulting taxonomic profiles quantified as percentages of the total microbiota were imported as R phyloseq object to facilitate visualisation and statistical comparisons [55]. To quantify Enterobacterales, we extracted the order of Enterobacterales from the MetaPhlAn output and labelled all other taxa ‘other’. Presence of Enterobacterales was defined as a relative abundance > 0%. Species richness and evenness were calculated using the R package ‘microbiome’, while Shannon diversity was calculated with the ‘vegan’ package.

### Resistome analysis

ARGs were detected using an assembly-based approach. Quality-trimmed reads were assembled into scaffolds using metaSPAdes (version 3.15.4, default parameters) [56]. Next, resistance genes were identified with ABRicate (version 0.8.13) using both the CARD (from 25 March 2021) and ResFinder (from 25 March 2021) databases, only retaining hits of full-length genes (100% coverage) with at least 97% identity. These criteria were selected based on visual inspection of the BLAST hits to balance high specificity and adequate sensitivity. As a control, we repeated the analyses using a coverage cut-off of 50% to include partial genes, which yielded equivalent results. ARGs were annotated with their respective target antibiotic and antibiotic class using the respective databases’ annotation files. Scaffolds were quantified by mapping the metagenomic reads back to the scaffolds using BWA-MEM (version 0.7.17) and samtools (version 1.10). Quantifications were normalised to reads per kilobase per million (RPKM) by dividing the number of reads mapped to each contig by the length of the contig and the number of high-quality reads used for the assembly, multiplied by 1000 * 1,000,000. To annotate scaffolds with additional information, scaffolds were taxonomically classified using the Genome Taxonomy Database Toolkit (GTDB-Tk; version 2.1.0) and the Contig Annotation Tool (CAT, version 5.2.3, parameters: ‘-r 10 -f 0.5’, [57] – which uses Prodigal version 2.6.3 [58]; DIAMOND version 2.0.6 [59]; and the NCBI BLAST nr database from 7 January 2021, https://ftp.ncbi.nlm.nih.gov/blast/db/), using CAT as primary annotation and filling in gaps in classification using the result of GTDB-Tk. The genomic origin of scaffolds with ARGs (chromosome or plasmid) was predicted using viralVerify (version 1.1, option ‘-p’, https://github.com/ablab/viralVerify) and we used only predictions that viralVerify reported as certain. All the scaffold annotation data was loaded into R (version 4.0.2; https://www.R-project.org/) for further analyses. Resistome richness was calculated by counting the number of different genes per sample, total abundance was calculated as the sum of all resistance genes’ abundance values (as RPKM) per sample, resistome Shannon diversity was calculated using the ‘vegan’ R package and Simpson evenness with the ‘microbiome’ package.

### Statistical analyses

The colonisation rate of MDR bacteria among patients was compared between pre- and short-term post-FMT and short-term and the long-term post-FMT using McNemar’s chi-square test for paired data (non-exact, without continuity correction). Depth of coverage of MDR bacteria in metagenomic data was compared between pre- and post-FMT with a paired *t*-test on log-transformed coverage values.

The effect of FMT on the colonisation rate of Enterobacterales in patients was tested using McNemar’s test without correction. Total abundances were compared using repeated measures ANOVA, followed by pairwise *t*-tests using Holm’s correction method.

For comparing taxonomic compositions of metagenomes and resistomes between donors and patients, we selected one value for each donor as representative. For principal component analyses (PCA), we picked the middle sample for each donor based on donation date (number of samples / 2, rounded up). Aitchison distance was used to calculate distances between microbiota or resistome compositions. Aitchison uses log-transformed values, which is impossible with zero, so we added pseudocounts. In PCA, donors and patients are compared using PERMANOVA and PERMDISP tests, considering the repeated measures in patients by using their ID as strata. Aitchison distances are compared using Wilcoxon rank sum tests. For comparisons of alpha diversity metrics using boxplots, we selected the median value as representative for each donor. Richness, total abundance, Shannon index, and Simpson evenness are compared between donors and patients using *t*-tests with Holm’s correction method. Abundance values were log-transformed. Within patients, all pre- and short-term post-FMT measures are compared using a paired *t*-test, while within the subgroup of 22 patients of whom we have collected long-term post-FMT samples values are first compared using repeated measures ANOVA. If *p* < 0.05, paired *t*-tests were used as post hoc test to determine differences between pre-FMT and long-term post-FMT and between short- and long-term post-FMT. Again, Holm’s correction method was used.

To evaluate if antibiotic (vancomycin) treatment duration before FMT influenced the resistome, we compared the pre-treatment duration of patients (*n* = 52) with their resistome richness (number of different ARGs), total abundance, Shannon diversity and Simpson evenness using Spearman correlation. All statistical tests were done in R version 4.0.2, using the base, rstatix, vegan, and pairwiseAdonis packages. A *p*-value below 0.05 was considered significant. Analysis scripts are available at Zenodo (https://zenodo.org/doi/10.5281/zenodo.10276220) [60].

## Results

### Donor and patient selection and population characteristics

During the sample collection period the NDFB provided faecal suspensions for 208 FMT treatments of 187 rCDI patients. From 87 patients (median age 73, interquartile range (IQR) 64–81 years, 56 females (64%)), we obtained stool samples from both pre- and short-term post-FMT to test for MDR bacteria by culture (Fig. 1). Twenty-two patients (median age 73, IQR 64–78 years; 14 females (64%)) provided a long-term post-FMT sample that was culture-tested. The median sampling times for patients are 1 day pre-FMT (IQR 1–3 days), 27 days post-FMT (IQR 20–48 days; short term), and 801 days post-FMT (IQR 447–1114 days; long term). Seventy-six donor samples from 15 different donors (median age 27, IQR 24–37.5; 9 females (60%)) were screened for MDR bacteria by culture. For shotgun metagenomic deep sequencing, we used 63 pairs of patient stool samples (patient median age: 73 years, interquartile range (IQR) 65–81 years; 40 females (63%)), 21 LTFU (one failed to provide a pre-FMT sample), and 70 donor stool samples from 11 different donors (median age 31 years, IQR 27–42 years; 6 females (55%); Fig. 1). The resistome analysis includes only complete sample triads (donor, pre-FMT and post-FMT), and sample tetrads with long-term post-FMT if both pre- and short-term post-FMT samples were available.

### Prevalence of multidrug-resistant bacteria decreases after FMT

**Fig. 2:**
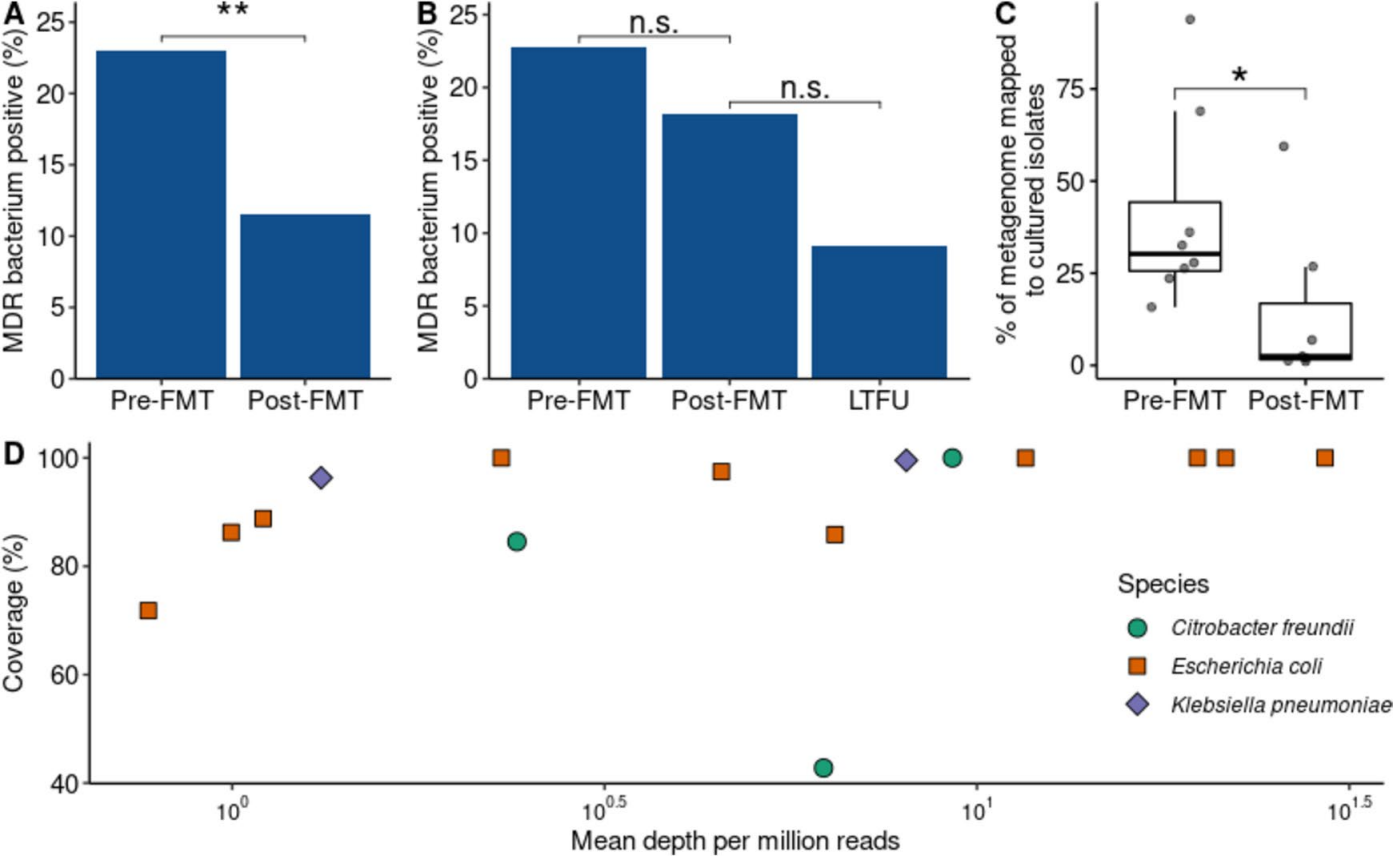
Effect of faecal microbiota transplantation on prevalence and abundance of cultured multidrug resistant bacteria. Stool samples of recurrent *C. difficile* infected (rCDI) patients were selectively cultured to assess the prevalence of multidrug-resistant (MDR) bacteria before and after faecal microbiota transplantation (FMT). We called samples MDR positive if at least one MDR bacterium was detected. Cultured isolates were subjected to whole-genome sequencing, and metagenomic sequencing data from the same stool samples were mapped to the assembled genomes to quantify the MDR bacteria in the metagenomes. **A** Prevalence of MDR bacteria in 87 rCDI patients. **B** Colonisation rates in 22 patients of whom long-term follow-up (~ 1–3 years after FMT) samples were collected. **C** Abundance of MDR bacteria based on metagenome data. **D** Breadth of coverage and relative abundance of MDR bacteria in metagenomic sequencing data per species. Asterisks indicate statistically significant differences, *: *p* < 0.05; **: *p* < 0.01; n.s.: not significant, MDR: multidrug resistant, FMT: faecal microbiota transplantation, LTFU: long-term follow-up

We began our study of the effect of FMT on MDR bacteria with selective cultures. Stool sample cultures of 15 donors and 87 patients pre- and post-FMT were assessed for carriership of MDR bacteria (Fig. 1). One donor had MDR bacterium-positive samples (1/15 = 6.7%), of which none were used for FMT. At least one MDR bacterium was detected in 20/87 (23.0%) of the patients before FMT (Fig. 2A, Table 1). Three weeks after FMT, the colonisation rate decreased to 10/87 (11.5%; *p* = 0.0075), of which 7 MDR bacteria were also detected before FMT. In the long term, the colonisation remained similar at 2/22 (9.1%; Fig. 2B; *p* = 0.16 compared to short-term post-FMT). Both MDR bacteria present in the LTFU were ESBL-producing *E. coli* also detected in the short-term post-FMT samples. Thereby, they appear to be long-term persisters. Within the subgroup of patients that provided long-term samples, there was no decrease in colonisation after FMT as we saw with the whole cohort (Fig. 2B; pre-FMT 5/22 = 22.7%, post-FMT 4/22 = 18.2%; *p* = 0.56). We compared data of MDR bacterial colonisation with CDI recurrence for a comprehensive analysis, but found that the numbers were too small to provide statistically meaningful results.

**Table 1:** Overview of cultured multidrug-resistant bacteria with genotype and phenotype

Patient	Sample timepoint	Species	Resistance phenotype	Genotype based on WGS	Detected in metagenome
P22	Post-FMT	*E. coli*	Aminoglycoside, fluoroquinolone, ampC	*APH(3’)-Ia, APH(6)-Id, APH(3″)-Ib, ANT(2″)-Ia, acrD, ampC, QnrB5, emrR*	Yes
P30	Post-FMT	*E. coli*	Aminoglycoside, fluoroquinolone	*acrD, emrR, emrD*	Yes
P31	Pre-FMT	*E. coli*	Aminoglycoside, fluoroquinolone, ceftazidime	*acdD, emrR, emrB, ampC*	Yes
P33	Pre-FMT	*K. pneumoniae*^a^	Aminoglycoside, fluoroquinolone, ESBL	*aadA2, aadA16, AAC(3-IId, TEM-1, SHV-119, CTX-M-14*	Yes
P33	Post-FMT	*K. pneumoniae*^a^	Aminoglycoside, fluoroquinolone, ESBL	*aadA2, aadA16, AAC(3-IId, TEM-1, SHV-119, CTX-M-14*	Yes
P38	Pre-FMT	*E. coli*^a^	Fluoroquinolone, ESBL	*CTX-M-27, ermR, emrB*	Yes
P38	Post-FMT	*E. coli*^a^	Fluoroquinolone, ESBL	*CTX-M-27, ermR, emrB*	Yes
P39	Pre-FMT	*E. coli*	Aminoglycoside, fluoroquinolone, ESBL	*CTX-M-15, OXA-1, acrD, AAC(3)-IIe, emrA, emrB, emrR*	Yes
P44	Pre-FMT	*C. freundii*^a^	Aminoglycoside, fluoroquinolone, ESBL	*CTX-M-15, OXA-1, AAC(3)-IIe, AAC(6’)-Ib-cr, APH(6)-Id, APH(3″)-Ib, QnrB6*	Yes
P44	Post-FMT	*C. freundii*^a^ + *E. coli*	Aminoglycoside, fluoroquinolone, ESBL + ESBL	*CTX-M-15, OXA-1, AAC(3)-IIe, AAC(6’)-Ib-cr, APH(6)-Id, APH(3″)-Ib, QnrB17*	Yes
P44	LTFU (3 yr)	*E. coli*	ESBL	NA	NA
P51	Pre-FMT	*C. freundii*	ESBL	*CTX-M-9*	Yes
P58	Pre-FMT	*E. coli*	Aminoglycoside, fluoroquinolone	*acrD, emrR*	Yes
P59	Pre-FMT	*E. coli*^a^	Aminoglycoside, fluoroquinolone, ESBL	*CTX-M-14, acrD, AAC(3)-IIe, APH(3″)-Ib, APH(6)-Id, emrR*	Yes
P59	Post-FMT	*E. coli*^a^	Aminoglycoside, fluoroquinolone, ESBL	*CTX-M-14, acrD, AAC(3)-IIe, APH(3″)-Ib, APH(6)-Id, emrR*	Yes
P59	LTFU (1 yr)	*E. coli*^a^	Aminoglycoside, fluoroquinolone, ESBL	NA	NA
P64	Pre-FMT	*E. coli*^a^	Aminoglycoside, fluoroquinolone	*AAC(6’)-Ib-cr, emrA, emrB, emrR*	NA
P64	Post-FMT	*E. coli*^a^	Aminoglycoside, fluoroquinolone	*acrD, APH(3")-Ib, APH(6)-Id, ampC, ampH, emrA, emrB, emrR*	NA
P65	Pre-FMT	*E. hormaechei_A (cloacae)*	Aminoglycoside, fluoroquinolone, ESBL	*ACT-27, CTX-M-15, OXA-1, TEM-1, AAC(3)-IIe, APH(6)-Id, APH(3")-Ib, AAC(6')-Ib-cr, QnrB6*	NA
P66	Pre-FMT	*M. morganii*	ESBL	*DHA-18*	NA
P67	Pre-FMT	*P. mirabilis*	ESBL	*CTX-M-1*	NA
P68	Pre-FMT	*P. mirabilis_B (vulgaris*/*mirabilis)*	ESBL	(none)^b^	NA
P69	Pre-FMT	*C. freundii*^a^	Aminoglycoside, fluoroquinolone, ESBL	*CTX-M-15, TEM-1, OXA-1, AAC(3)-IIe, APH(3″)-Ib, APH(6’)-Id, QnrB6*	NA
P69	Post-FMT	*C. freundii*^a^	Aminoglycoside, fluoroquinolone, ESBL	*CTX-M-15, TEM-1, OXA-1, AAC(3)-IIe, APH(3″)-Ib, APH(6’)-Id, QnrB17*	NA
P70	Pre-FMT	*E. coli*^a^	ESBL	*ampC, ampH, SHV-134*	NA
P70	Post-FMT	*E. coli*^a^	ESBL	*ampC, ampH, SHV-134*	NA
P71	Pre-FMT	*K. pneumoniae*	ESBL	NA	NA
P72	Pre-FMT	*P. hauseri*	ESBL	NA	NA
P73	Pre-FMT	*C. freundii*	ESBL	NA	NA
P74	Pre-FMT	*E. cloacae*	ESBL	NA	NA
P75	Pre-FMT	*E. cloacae*^a^	ESBL	NA	NA
P75	Post-FMT	*E. cloacae*^a^	ESBL	NA	NA

*FMT* Faecal microbiota transplantation, *LTFU* Long-term follow-up, *ESBL* Extended-spectrum beta-lactamase, *NA* Data not available (because the isolate and/or the metagenome were not sequenced), *WGS* Whole-genome sequencing. Species names are listed as in the Genome Taxonomy Database (GTDB), and the alias known by the National Center for Biotechnology Information (NCBI) is given in parentheses when different. When multiple multidrug-resistant bacteria were cultured from the same stool, isolate characteristics are separated by a plus (‘ + ’) sign

^a^Same species before and after FMT, persistence is likely based on resistance genotype (Additional file 1: Fig. S1-3) when available

^b^No antibiotic resistance genes were detected in the genome sequence data

### Whole-genome sequencing of multidrug-resistant and comparison with metagenomics reveals that MDR bacteria had higher abundances in rCDI patients before FMT than after FMT

Next, we checked the resistance genotype of isolates using WGS and combined the isolate data with faecal metagenomics to quantify the abundance of MDR bacteria in the gut microbiota. Twenty-four cultured isolates of multidrug-resistant bacteria were subjected to WGS. In all but one genome, we were able to detect ARGs associated with the resistance phenotype; e.g., ESBL genes in isolates classified as ESBL-producing (Table 1; Additional file 1: Fig. S1-3). Furthermore, we mapped metagenomic reads to the assembled isolate genome to compare essay sensitivity and determine relative abundances in the microbiota. As expected in patients pre-treated with antibiotics, we found that MDR bacteria had higher abundances in rCDI patients before FMT than after FMT (Fig. 2C; *p* = 0.0159). We detected near-complete genomes of MDR isolates in the metagenomes with various depths (Fig. 2D). Only one *Citrobacter freundii* genome was covered less than half (43%) in the metagenome. We then compared resistance genes detected in the WGS data to those detected in metagenomic data to estimate the sensitivity of metagenomic sequencing compared to culturing. These resistance genes of cultured isolates were also found in their respective metagenomes (Table 1). Besides, metagenomic data from patient P44 suggested the presence of an ESBL-producing *E. coli* in the pre-FMT sample, while culture only picked it up in the post-FMT faeces. These data suggest that combining bacterial culture with metagenomic sequencing can be used synergistically and provide more detailed results than either method alone. In summary, we find that both the prevalence and the abundance of MDR bacteria were decreased after FMT.

### FMT makes gut microbiota more donor-like and decreases Enterobacterales, also in long term

**Fig. 3:**
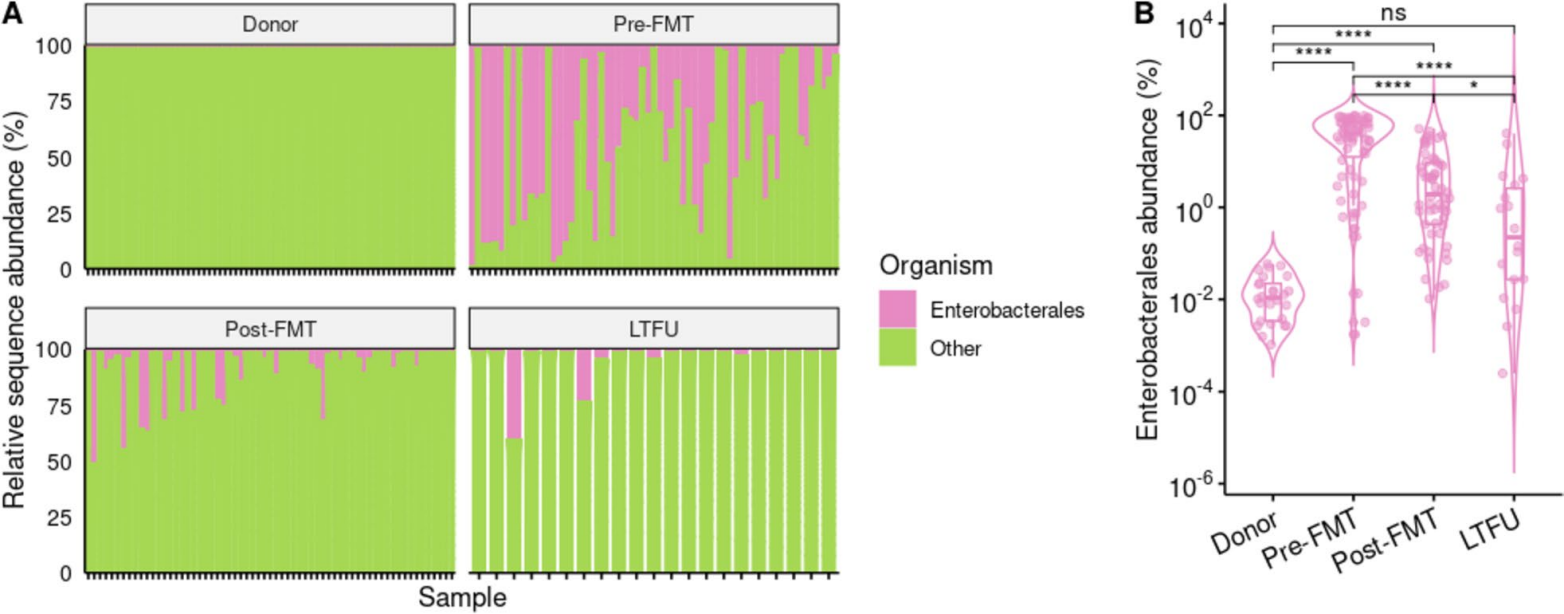
Prevalence and abundance of Enterobacterales in faecal donors and faecal microbiota transplantation recipients.** A** Relative abundances of Enterobacterales in metagenomes as determined by MetaPhlAn4. **B** Total abundances of Enterobacterales in stool donors and rCDI patients treated with FMT sampled one day before (Pre) FMT, 3 weeks after (Post) FMT and 1–3 years after FMT (long-term follow-up, LTFU). Statistically significant differences are indicated by asterisks, *: *p* < 0.05; ****: *p* < 0.0001. FMT: faecal microbiota transplantation, LTFU: long-term follow-up

To gain deeper understanding in how FMT affected the gut microbiota in this cohort, we profiled faecal metagenomes of donors and recipients using MetaPhlAn4. Donors had microbiota dominated by Bacillota (formerly Firmicutes), Bacteroidota (formerly Bacteroidetes), and Actinomycetota (formerly Actinobacteria; Additional file 1: Fig. S4A). Enterobacterales were present in 26/70 donor stools (37%) at ~ 0.01% abundance (Fig. 3A, B). In rCDI patients, that underwent anti-CDI treatment prior to FMT (53 × vancomycin, 6 × fidaxomicin, 1 × metronidazole, 1 × metronidazole + vancomycin, 2 unknown), Actinomycetota and Bacteroidota were much less present, while Proteobacteria (mostly *Escherichia coli* or *Klebsiella pneumoniae*) were often dominant (> 50% abundance in 31/63 patients = 49%; Additional file 1: Fig. S4B). Enterobacterales were present in all pre-FMT patient stools (Fig. 3A). Shortly after FMT, the prevalence of Enterobacterales dropped to 58/63 (92%; *p* = 0.0253) and the abundance decreased as well (Fig. 3B; adjusted *p* < 0.0001). In the longer term after FMT, the prevalence of Enterobacterales did not change (18/21 = 86%; *p* = 0.655 compared to 3 weeks post-FMT), but the abundance decreased further (adjusted *p* = 0.025), and was no longer distinct from the donors’ (*p* = 0.09).

**Fig. 4:**
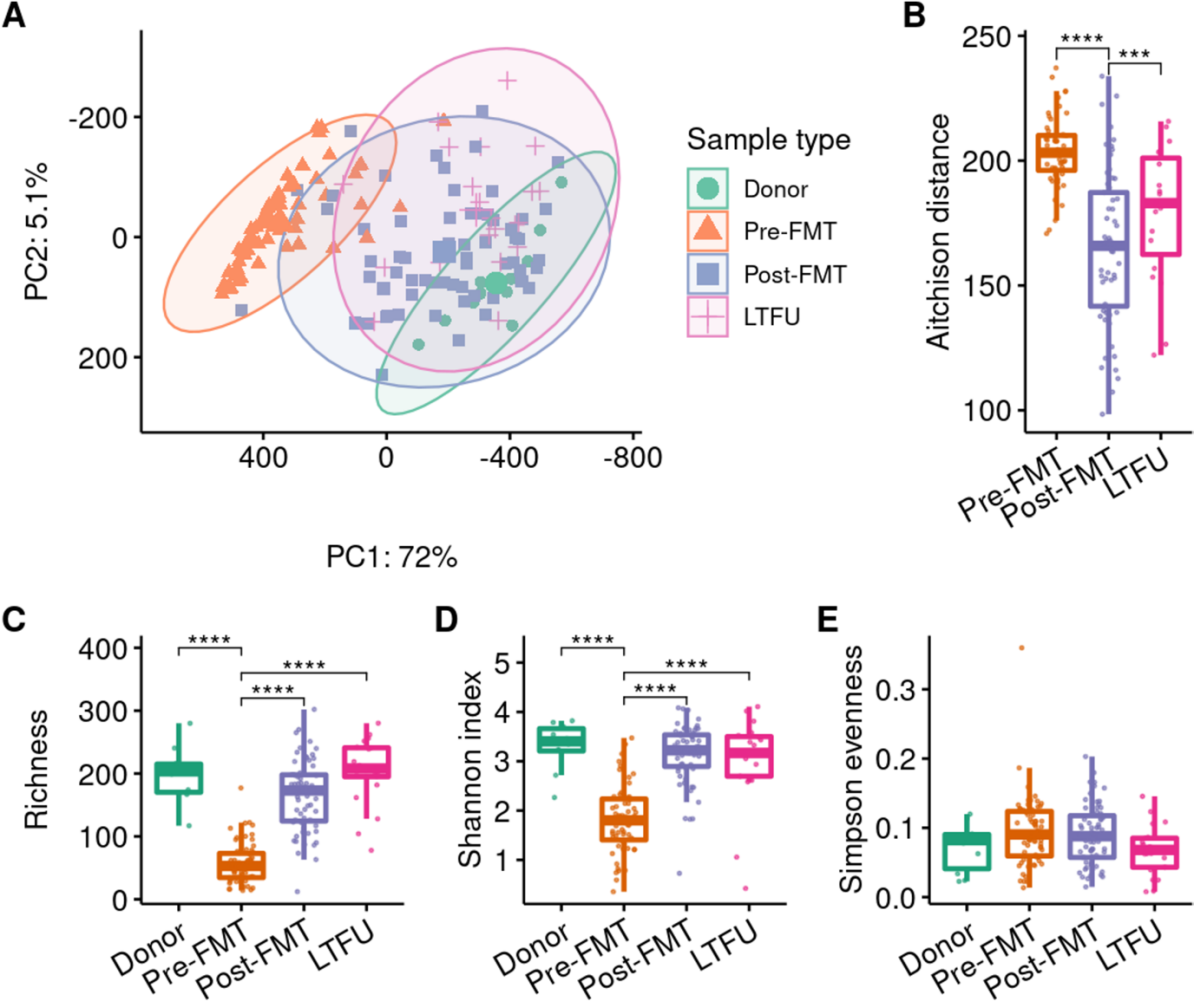
Comparison of gut microbiota composition and diversity. Species composition of metagenomes was determined by MetaPhlAn4. **A** Beta diversity expressed as Aitchison distances in a principal component analysis (PCA). Percentages on the *X*- and *Y*-axis represent the variance explained by the first two components. **B** Aitchison distance from patient species profile to corresponding donor. **C**–**E** Species richness, Shannon index and Simpson evenness compared between donors and recipients, respectively. Asterisks indicate statistically significant differences, ****: *p* < 0.0001; FMT: faecal microbiota transplantation, LTFU: long-term follow-up

We further studied the species composition of donor and patient metagenomes using PCA (Fig. 4A). Patient species composition was different from the donors’ at all timepoints (PERMANOVA: overall *p* = 0.001, pre-FMT *p* = 0.003, post-FMT *p* = 0.014, LTFU *p* = 0.014; PERMDISP: *p* < 0.0001). However, after FMT the patients’ microbiota were more similar to their donors’ (Fig. 4B; *p* < 0.0001). This shift in microbiota appears at least partly attributable to the FMT and is not solely due to recovery after antibiotic use, as indicated by a comparison between patient post-FMT species compositions to those of the donor that was used against unrelated donor samples (Additional file 1: Fig. S5; *p* < 0.0001). In the long term, species compositions in FMT recipients moved away from the donors’ (post-FMT vs LTFU adjusted *p* = 0.0008), but was still more donor-like than before FMT (adjusted *p* = 0.002).

We then compared the alpha diversity between species profiles of donor and patient metagenomes. Species richness and Shannon diversity were higher in donors than in rCDI patients before FMT (Fig. 4C, D; adjusted *p* < 0.0001) and increased dramatically in patients after FMT (adjusted *p* < 0.0001) to levels as seen in donors (adjusted *p* > 0.1). Richness and Shannon index remained high at the long term. The Simpson evenness, also known as inverse Simpson index or Simpson’s dominance, was not different between donors and patients (Fig. 4E; adjusted *p* > 0.3). Overall, our data show the expected pattern of lower diversity in rCDI patients, high diversity in FMT donors, and increased diversity in patients after FMT. After FMT, both the prevalence and abundance of Enterobacterales were decreased in patients.

### FMT decreases abundance of resistance genes, but not their diversity

**Fig. 5:**
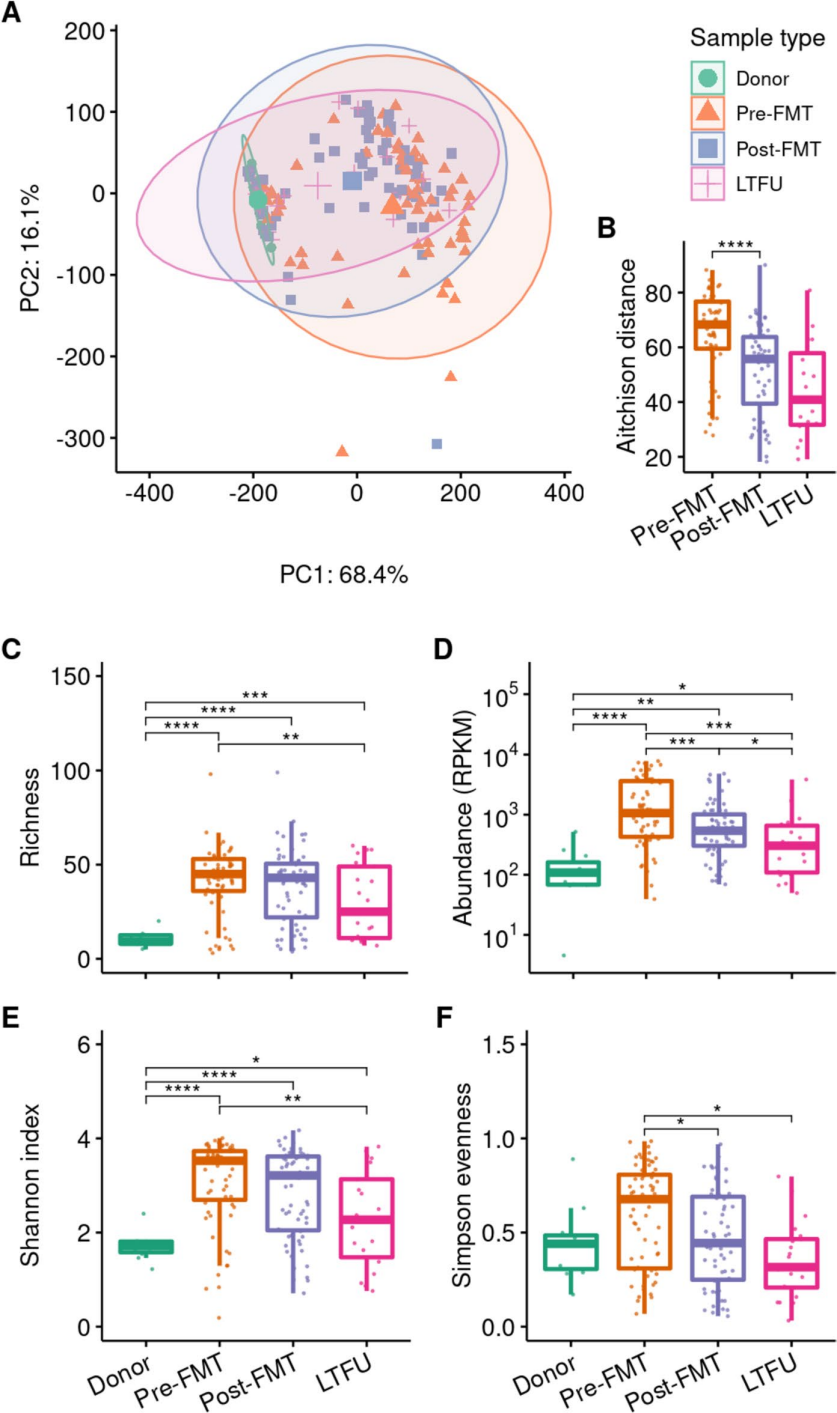
Overview of resistomes of faeces donors and faecal microbiota transplantation recipients.** A** Principal component analysis (PCA) of resistomes, based on Aitchison distances. Percentages on the *X*- and *Y*-axis represent the variance explained by the first two components. **B** Aitchison distance from patient antibiotic resistance gene profiles to corresponding donor. **C**–**F** Antibiotic gene richness, total abundance, Shannon index and Simpson evenness compared between groups and between recipient timepoints, respectively. Asterisks indicate statistically significant differences, *: *p* < 0.05; **: *p* < 0.01; ***: *p* < 0.001; ****: *p* < 0.0001. FMT: faecal microbiota transplantation, LTFU: long-term follow-up

Using the same metagenomic sequencing data, we determined the resistome using a custom assembly-based approach. We quantified differences in resistome composition between donors and patients using PCA (Fig. 5A). Donors had similar resistomes and often had the same ARGs for aminoglycoside, diaminopyrimidine and tetracycline resistance (Additional file 1: Fig. S6), while rCDI patients had a very different resistome (PERMANOVA, *p* = 0.003; PERMDISP, *p* < 0.0001), in which different ARGs for beta-lactam and fluoroquinolone resistance as well as multidrug efflux pumps were prevalent (Additional file 1: Fig. S6-7). After FMT, a shift in the patients’ resistome towards a donor-like composition is visible (Fig. 5B; adjusted *p* < 0.0001), although it remained different from the donors’ (p = 0.003). At long-term follow-up, the resistome was neither more nor less donor-like than at 3 weeks after FMT (Fig. 5B; adjusted *p* = 0.123) and was still statistically different from the donors’ (*p* = 0.012). These differences in resistome composition between donors and patients and the shift after FMT are also visible when viewing the resistome as relative abundances per antibiotic class (Additional file 1: Fig. S8).

We find that patients before FMT had more different resistance genes (higher resistome richness) in their faecal metagenomes than donors (adjusted *p* < 0.0001; Fig. 5C). The duration of vancomycin pre-treatment did not significantly influence the resistome (Additional file 1: Fig. S9). After FMT, resistome richness in patients did not change (adjusted *p* > 0.1) and remained higher than in donors (short-term post-FMT: adjusted *p* < 0.0001; long-term: adjusted *p* = 0.0002). The total abundance of resistance genes was also higher in patients pre-FMT than in donors (Fig. 5D; adjusted *p* < 0.0001), but in contrast to the resistome richness, abundance decreased in patients shortly after FMT (*p* = 0.0003). In the long term, the abundance lowered further (adjusted *p* = 0.02), although abundances remained higher than in donors (adjusted *p* = 0.02). The Shannon index combines richness and abundance and likewise showed a higher resistome diversity in rCDI patients compared to donors, and a decrease after FMT (Fig. 5E). The Simpson evenness shows no statistical difference between donors and patients (Fig. 5F; adjusted *p* > 0.1), but indicates a decrease of resistome diversity in patients after FMT (*p* = 0.017). In summary, FMT appears to alter the diversity of the resistome in recipients by lowering relative abundances of ARGs.

We observed different prevalence and abundance patterns of ARGs from different antibiotic classes (Additional file 1: Fig. S6-8). To explore this further, we selected classes of which genes were present in both donors and patients and divided them in two groups. One group (beta-lactamase, fluoroquinolone and multidrug efflux pump) consists of genes that are rare in donors and common and abundant in patients (Additional file 1: Fig. S10 A-C and G-I). The abundance of genes in this group decreased shortly after FMT, while the resistome richness decreased only in the long term. The second group (aminoglycoside, diaminopyrimidine and tetracycline) is common in donors (Additional file 1: Fig. S10D-F and J-L). Genes from this donor-associated group may have been transferred to the recipients, resulting in greater resistome richness after FMT and their abundance did not decrease after FMT. These results highlight that the effects of FMT on the resistome vary depending on type of antibiotic and the taxa that carry the genes.

### Remarkable resistances

We found a number of ESBL genes in our resistome data, and also in donor faeces. Furthermore, we found carbapenamase genes and one colistin resistance gene (mrc-10_1, predicted to be on a plasmid) only in rCDI patients before FMT. Vancomycin genes were detected by metagenomics in 7 out of 63 patients before FMT (11.1%) and 11 / 63 after FMT (17.5%; Additional file 1: Fig. S11). Besides, our cultures picked up a vancomycin-resistant *Enterococcus faecalis* from a post-FMT stool, which was penicillin-susceptible and therefore not listed as MDRO. These resistances as well as those predicted to be on plasmids are discussed in more detail in the supplementary results and Figures S11, S12, and S13 (Additional file 2; Additional file 1: Fig. S11-13).

### Predicted plasmid-mediated antibiotic resistance remains high

**Fig. 6:**
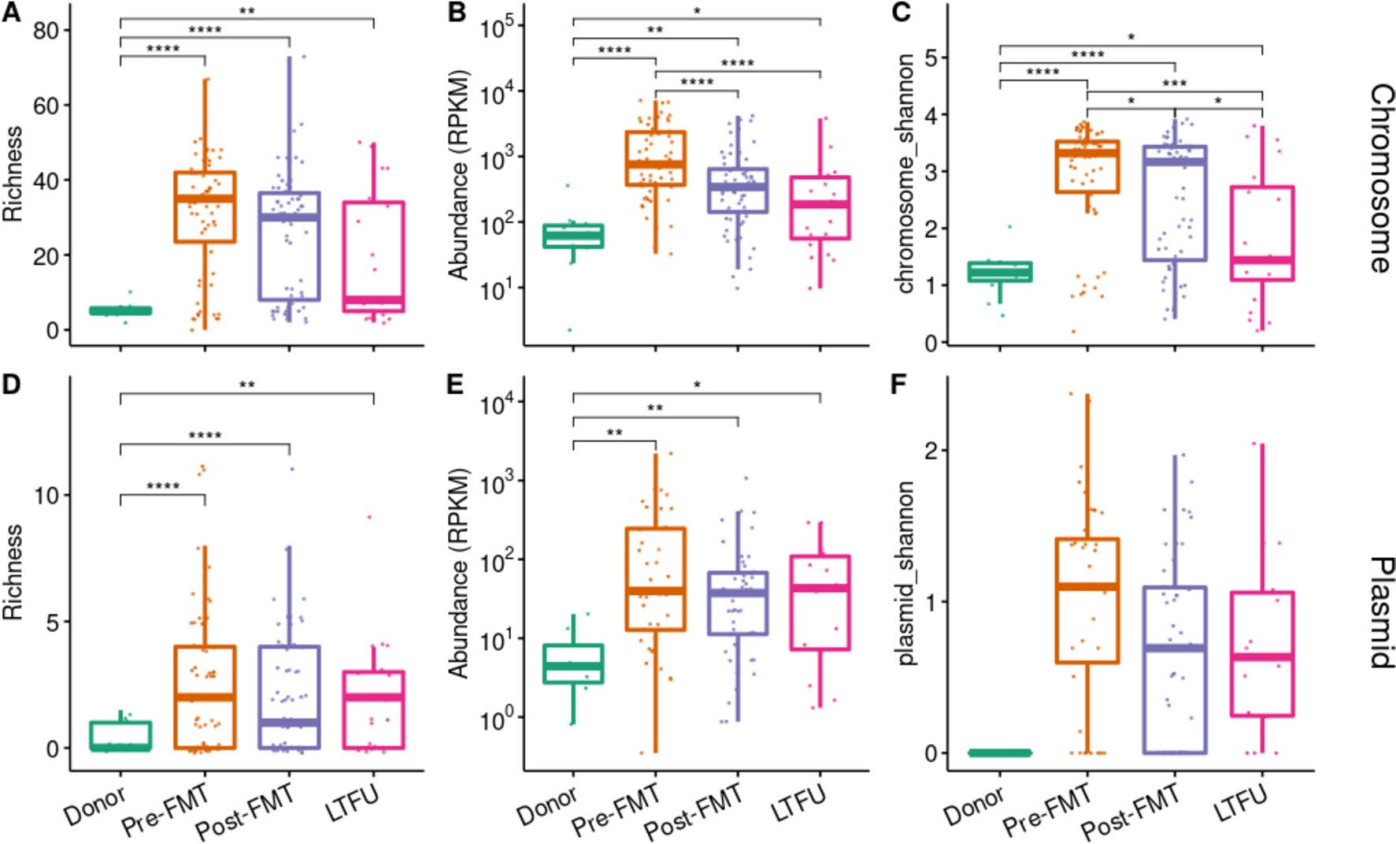
Resistome comparisons for chromosomal resistance genes and plasmid-associated resistance genes. Antibiotic gene-carrying scaffolds’ were predicted to derive from chromosomes or plasmids using viralVerify. **A** Resistome richness, **B** Total abundance, and **C** Shannon index of scaffolds predicted to be chromosomal. **D**–**F** Same parameters for scaffolds predicted to derive from plasmids. Statistically significant differences are indicates by asterisks, *: *p* < 0.05; **: *p* < 0.01; ***: *p* < 0.001; ****: *p* < 0.0001. FMT: faecal microbiota transplantation, LTFU: long-term follow-up

We hypothesised that the relative persistence of the resistome after FMT may be linked to plasmids. To test this, we used the plasmid prediction algorithm from viralVerify and assessed which contigs with resistance genes were likely to derive from chromosomes and which from plasmids. Most (4567 / 6662 or 68.6%) of the resistance genes were predicted to derive from chromosomes and 400 (6%) likely derived from plasmids. The remaining 1695 (25.4%) contigs with ARGs could not confidently be classified to either plasmid or chromosome. Unlike chromosomal resistances, which follow the general resistome pattern and decrease after FMT (Fig. 6A–C), we find that the resistome richness, abundance, and diversity of ARGs derived from plasmids were higher in rCDI patients than in donors and stayed higher after FMT (adjusted *p* ≤ 0.01; Fig. 6D–F). This effect persisted up to 3 years after the FMT, suggesting that FMT may not significantly influence plasmid-mediated antibiotic resistance.

## Discussion

Our current study leverages the strengths of bacterial culture techniques and metagenomic sequencing to provide a comprehensive view of antibiotic-resistant bacteria in the intestinal tract in the weeks and years after FMT. We find that FMT decreased prevalence and abundance of MDR bacteria and led to more diverse and donor-like microbiota and ARG compositions. However, the resistome of patients stayed different from the donors’ even in the 1–3-year follow-up. This study provides a unique insight into the long-term effects of FMT on the resistome of rCDI patients, although the follow-up is limited in the number of responders and variable time after FMT. Whether there is a correlation of persistence of antibiotic resistance with CDI recurrence could not be assessed given the limited number of relapses in our cohort.

For the interpretation of the resistome of recipients after FMT, we need to consider the characteristics of the recipients’ resistome before transplant, and of the donor resistome. Our patient cohort consists of mostly elderly people with significant comorbidity and disturbance of the gut microbiota by multiple courses of antibiotic treatment for recurrent *C. difficile* infections. This has been described to alter the gut resistome [61]. Contrastingly, the gut of healthy individuals harbours mainly anaerobic commensal bacteria, which frequently carry aminoglycoside and tetracycline resistance genes [62]. Our results indicate that these classes of antibiotic resistance were indeed common in stool donors, and also in rCDI patients. The observed shift in the recipients’ resistome composition after FMT is likely a reflection of the introduction of the anaerobic commensal bacteria, resulting in different effects of FMT on various antibiotic classes [63]. Some ARGs and predicted plasmids persisted in the recipients after FMT and, therefore, the resistome composition remained different from the donors’.

While we have no data on the effect of FMT on the prevalence of infections with MDR bacteria post-FMT, in other patient groups it has been found that FMT can decrease the risk of infections [64, 65], or delay the development of MDR infections [34]. Assuming that MDR bacteria are not eradicated after FMT, there may still be a risk of infection when the intestinal environment becomes hospitable for outgrowth of MDR bacteria due to, for example, antibiotics [14–16]. Nonetheless, FMT may reduce the number of infections with MDR bacteria even if the patients’ guts are not decolonised with MDR bacteria [66]. The hypothesised mechanism is that the gut microbiota is restored by FMT to a balanced state that is resilient to MDR carrying bacteria [64], for example by nutrient competition [67, 68], restoring short-chain fatty-acid production [69, 70] and production of bacteriocins [71]. This situation may be described as reduced infection susceptibility or infection resistance. Our data give new details on how the taxonomic composition of the microbiota may give shape to reduced MDR infections.

Antibiotic treatment not only affects bacteria, but may also cause fungi to proliferate [72–74]. We recently found using ITS2 sequencing that CDI patients have increased abundances of *Candida* spp., and decreased *Aspergillus* spp. and *Penicillium* spp. compared to controls [75], but the shotgun metagenomic approach in our present study is not adequate to detect fungi with a median relative abundance of 0.003% detected in 5 samples. Thus, to further elucidate the role of the mycobiome, and the fungal resistome, in relation to FMT more targeted experiments are needed.

Our study of the resistome is limited by the annotation of ARGs in publicly available databases and prediction tools for cellular localisation (chromosome or plasmid-based) and do not allow us to definitively link ARGs to specific species. Furthermore, the method we used does not include chromosomal point mutations that confer resistance to antibiotics, which can often be linked to species. Future high-throughput bacterial cultivation efforts will shed light on previously uncharacterised ARGs, what species carry them and on what sort of genetic element [76]. These improved culturomics methods from diverse environments can in turn help alleviate the biases in genome databases, in which Gram-negative pathogens such as Enterobacterales have been relatively overrepresented. A recent large-scale analysis pointed out that the clinically most relevant ARGs are restricted to particular taxa, most notable Enterobacterales and Bacteroides [77]. Replacement of antibiotic-resistant bacteria may require introduction of susceptible strains [34], which, combined with our data, may suggest a role for FMT donors carrying antibiotic-susceptible Enterobacterales. While FMT may reduce the number or eliminate specific bacteria, horizontal gene transfer may lead to persistence of ARGs when they are transferred to persisting or newly acquired bacteria. Plasmids are potentially mobile genetic elements that facilitate transfer of DNA between bacteria. Plasmid-mediated antibiotic resistance is a growing problem worldwide and is especially associated with Enterobacterales [78, 79]. We find, however, that most antibiotic resistance genes in the gut microbiota are predicted to be chromosomally encoded, though we cannot exclude the possibility that these ARGs are located on mobile genetic elements. To assess the mobility of resistance genes, techniques are needed that can link ARGs to their host organism, such as Meta-HiC [62] or OIL-PCR [80].

## Conclusions

Our study points towards possibilities and limitations of the use of FMT for the eradication of MDR bacteria in the gut. Based on pre- and post-FMT resistome analysis (including a unique LTFU of 1–3 years), we find that FMT induces significant changes in the recipient resistome, that may be associated with a reduction in the abundance of Enterobacterales. However, we also find that specific recipient-ARGs persist. The clinical consequences of this persistence were not included in this study and require further analyses in large cohort of FMT-treated patients. To better assess the possible benefits in MDR eradication, we need larger (randomised controlled) trials and multi-omics studies combined with classical microbiological methods that can link ARGs to bacterial taxa, and to the host’s gut ecosystem. Additionally, the use of local, national and international registries for FMT can help collect long-term data to assess infection risks in different patient populations [81, 82]. Besides keeping track of MDR-related outcomes, these registries facilitate evaluation of other long-term microbiota-related risks, such as CDI recurrence or procarcinogenic bacteria [49, 83, 84]. Finally, studies with control patients and more diverse patients are needed to explain the resistome differences and obtain more generalisable results. This will pave the way for evaluating the feasibility of FMT to control antibiotic resistance in infection-susceptible patients.

### Supplementary Information

**Additional file 1: Fig. S1.** Detected antibiotic resistance genes in 5 *Escherichia coli* isolates resistant to aminoglycosides and fluoroquinolones. **Fig. S2.** Detected antibiotic resistance genes in extended-spectrum beta-lactamase producing *Escherichia coli* and *Citrobacter freundii* isolates. **Fig. S3.** Detected antibiotic resistance genes in extended-spectrum beta-lactamase producing *Enterobacter hormaechei_A*, *Klebsielle pneumoniae*, *Morganella morganii* and *Proteus mirabilis* isolates. **Fig. S4.** Taxonomic composition of faecal metagenomes of FMT donors and recipients. **Fig. S5.** Aitchison distance from 63 post-FMT recipients’ species composition to 8 used and unrelated donors. **Fig. S6.** Occurrence and abundance of antibiotic resistance genes in faecal metagenomes of FMT donors and recipients. **Fig. S7.** Occurrence and abundance of antibiotic resistance genes of other classes in faecal metagenomes of FMT donors and recipients. **Fig. S8.** Resistome composition as relative abundance of antibiotics classes. **Fig. S9.** Resistome parameters compared with duration of vancomycin pre-treatment in days. **Fig. S10.** Richness and abundance of antibiotic genes of selected classes. **Fig. S11.** Antibiotic resistance genes of high clinical importance. **Fig. S12.** Overview of antibiotic resistance genes predicted to be on plasmids (part 1/2). **Fig. S13.** Overview of antibiotic resistance genes predicted to be on plasmids (part 2/2).

**Additional file 2: Supplementary results**. Additional information regarding the detection of antibiotic resistance genes encoding carbapenemases, ESBL, and colistin and vancomycin resistance.

## Data Availability

Sequencing reads generated for this study are available in the European Nucleotide Archive under project numbers PRJEB64622 (https://www.ebi.ac.uk/ena/browser/view/PRJEB64622) [48], PRJEB44737 (https://www.ebi.ac.uk/ena/browser/view/PRJEB44737) [50], and PRJEB64621 (https://www.ebi.ac.uk/ena/browser/view/PRJEB64621) [51]. Code to reproduce analyses and generate figures are available at Zenodo (https://doi.org/10.5281/zenodo.10276220) [60].

## Abbreviations

CDI: *Clostridioides difficile* Infection
FMT: Faecal microbiota transplantation
rCDI: Multiple recurrent *C. difficile* infection
MDR: Multidrug-resistant
ESBL: Extended-spectrum beta-lactamase
RCT: Randomised controlled trial
ARG: Antibiotic resistance gene
NDFB: Netherlands Donor Feces Bank
LTFU: Long-term follow-up
VRE: Vancomycin-resistant *Enterococcus*
MRSA: Methicillin-resistant *Staphylococcus aureus*
MALDI-TOF: Matrix-assisted laser desorption ionisation-time of flight
WGS: Whole-genome sequencing
RPKM: Reads per kilobase per million
ENA: European Nucleotide Archive
PCA: Principal component analysis
ANOVA: Analysis of variance
IQR: Interquartile range
PERMANOVA: Permutational analysis of variance
PERMDISP: Permutational analyses of multivariate dispersions
