# Supplementary material for: Long-term beneficial effect of faecal microbiota transplantation on colonisation of multidrug-resistant bacteria and resistome abundance in patients with recurrent Clostridioides difficile infection
Source: Genome Med. 2024 Feb 28;16:37. doi: 10.1186/s13073-024-01306-7 (PMC10902993; doi:10.1186/s13073-024-01306-7)
Supplement: Supplementary file 1 — Additional file 1: Fig. S1. Detected antibiotic resistance genes in 5 Escherichia coli isolates resistant to aminoglycosides and fluoroquinolones. Fig. S2. Detected antibiotic resistance genes in extended-spectrum beta-lactamase producing Escherichia coli and Citrobacter freundii isolates. Fig. S3. Detected antibiotic resistance genes in extended-spectrum beta-lactamase producing Enterobacter hormaechei_A, Klebsielle pneumoniae, Morganella morganii and Proteus mirabilis isolates. Fig. S4. Taxonomic composition of faecal metagenomes of FMT donors and recipients. Fig. S5. Aitchison distance from 63 post-FMT recipients’ species composition to 8 used and unrelated donors. Fig. S6. Occurrence and abundance of antibiotic resistance genes in faecal metagenomes of FMT donors and recipients. Fig. S7. Occurrence and abundance of antibiotic resistance genes of other classes in faecal metagenomes of FMT donors and recipients. Fig. S8. Resistome composition as relative abundance of antibiotics classes. Fig. S9. Resistome parameters compared with duration of vancomycin pre-treatment in days. Fig. S10. Richness and abundance of antibiotic genes of selected classes. Fig. S11. Antibiotic resistance genes of high clinical importance. Fig. S12. Overview of antibiotic resistance genes predicted to be on plasmids (part 1/2). Fig. S13. Overview of antibiotic resistance genes predicted to be on plasmids (part 2/2). [file 13073_2024_1306_MOESM1_ESM.docx]

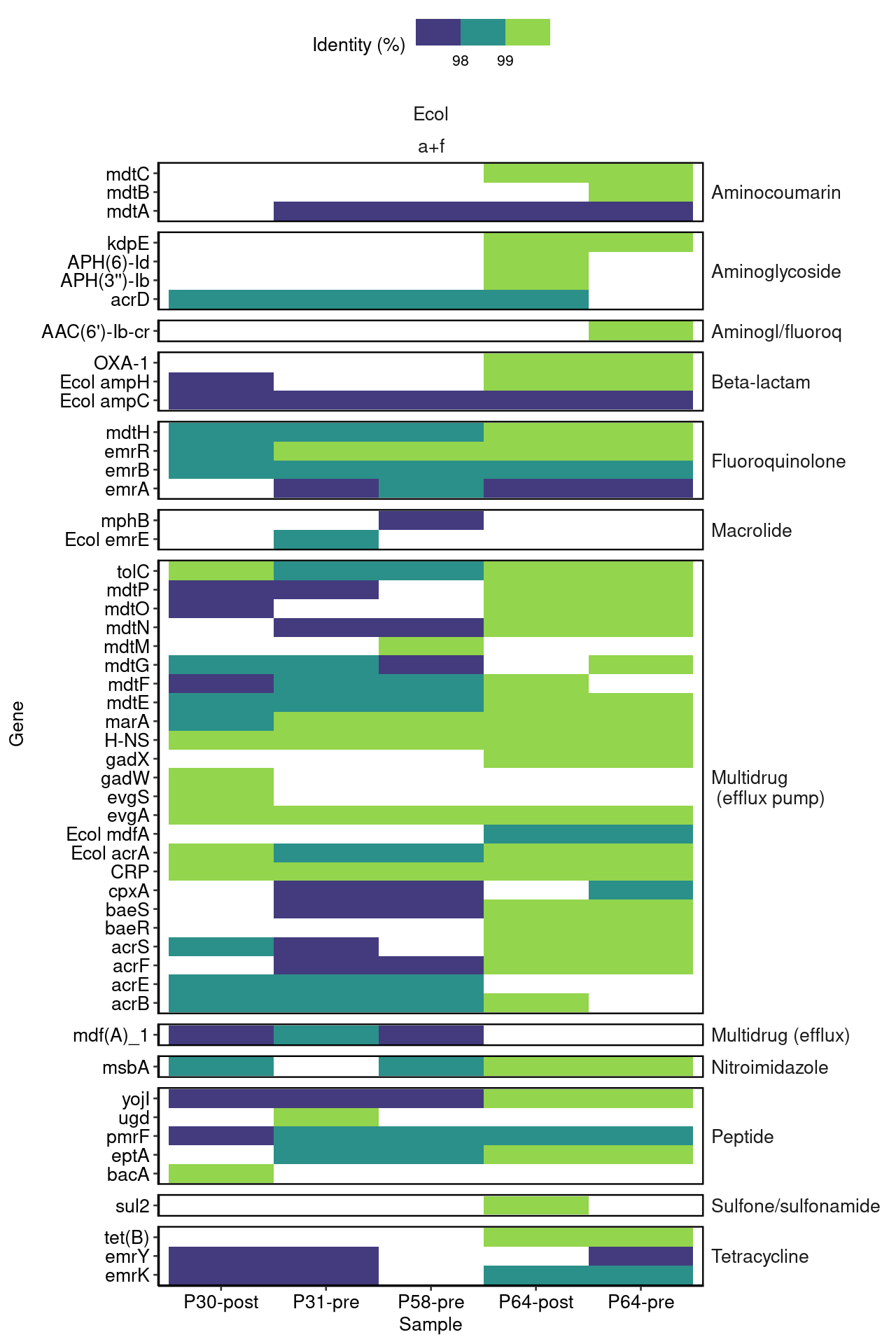


**Fig. S1. Detected antibiotic resistance genes in 5 *Escherichia coli* isolates resistant to aminoglycosides and fluoroquinolones.** We used broth-enriched cultures to detect multidrug resistant bacteria in stool samples of recurrent *C. difficile* infected (rCDI) patients before (n=3) and after (n=2) faecal microbiota transplantation (FMT). The bacterial isolates were then subjected to whole-genome sequencing, after which we screened the genomes for resistance genes using ABRicate with the CARD and ResFinder databases. The figure shows antibiotic genes grouped by antibiotic class, colours represent nucleotide BLAST identity.
Ecol: *Escherichia coli*, a+f: aminoglycoside and fluoroquinolone resistant, pre: sample collected the day before FMT, post: sample collected ~3 weeks after FMT


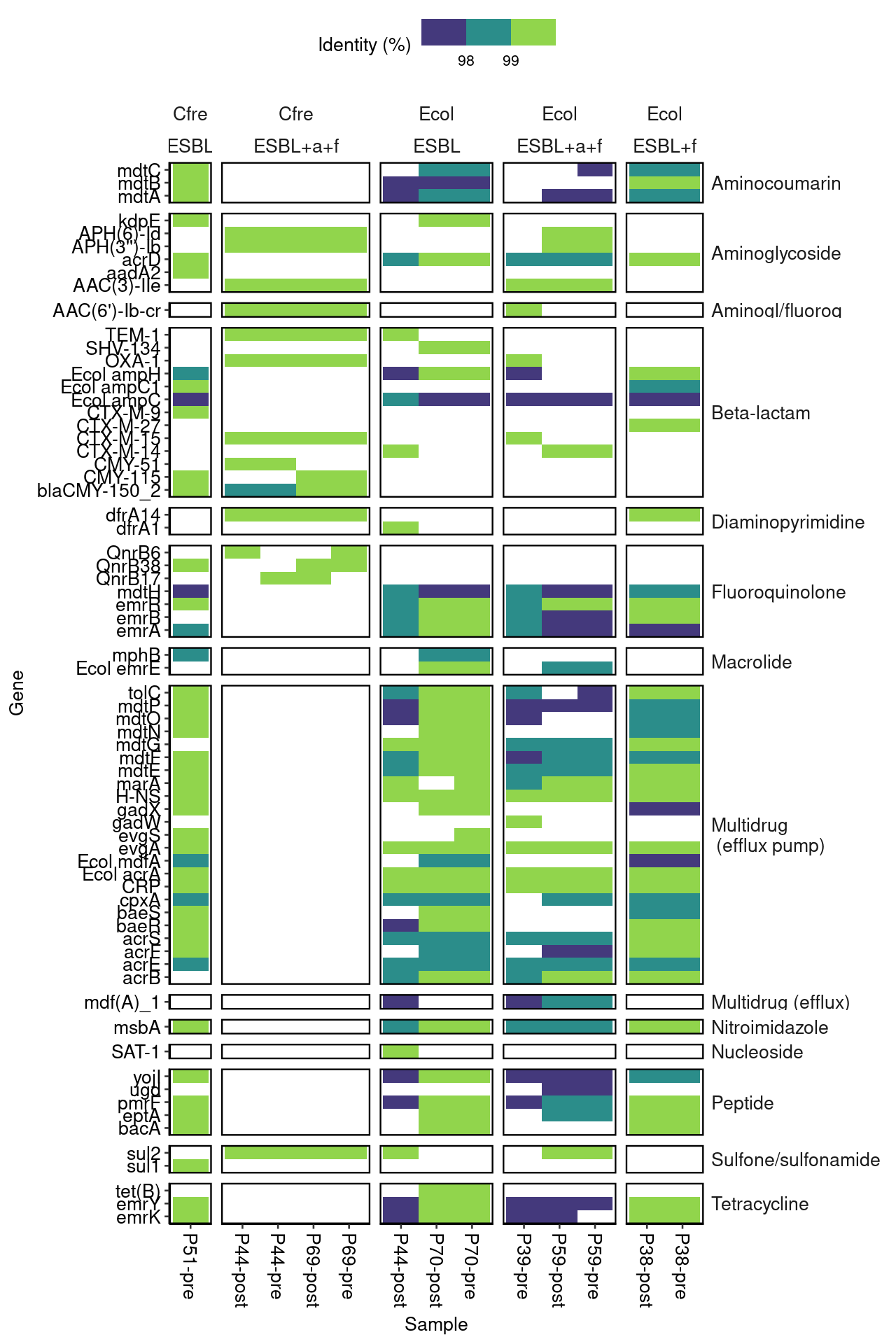


**Fig. S2. Detected antibiotic resistance genes in extended-spectrum beta-lactamase producing *Escherichia coli* and *Citrobacter freundii* isolates**. Three *C. freundii* isolates were cultured from pre-FMT samples, 2 from post-FMT; 4 *E. coli* isolates were from pre-FMT samples and 4 from post-FMT. Summarised by antibiotic class, colours represent nucleotide BLAST identity.
Cfre: *Citrobacter freundii*, Ecol: *Escherichia coli*, ESBL: extended-spectrum beta-lactamase-producing, a: aminoglycoside- resistant, f: fluoroquinolone-resistant, pre: sample collected the day before FMT, post: sample collected ~3 weeks after FMT


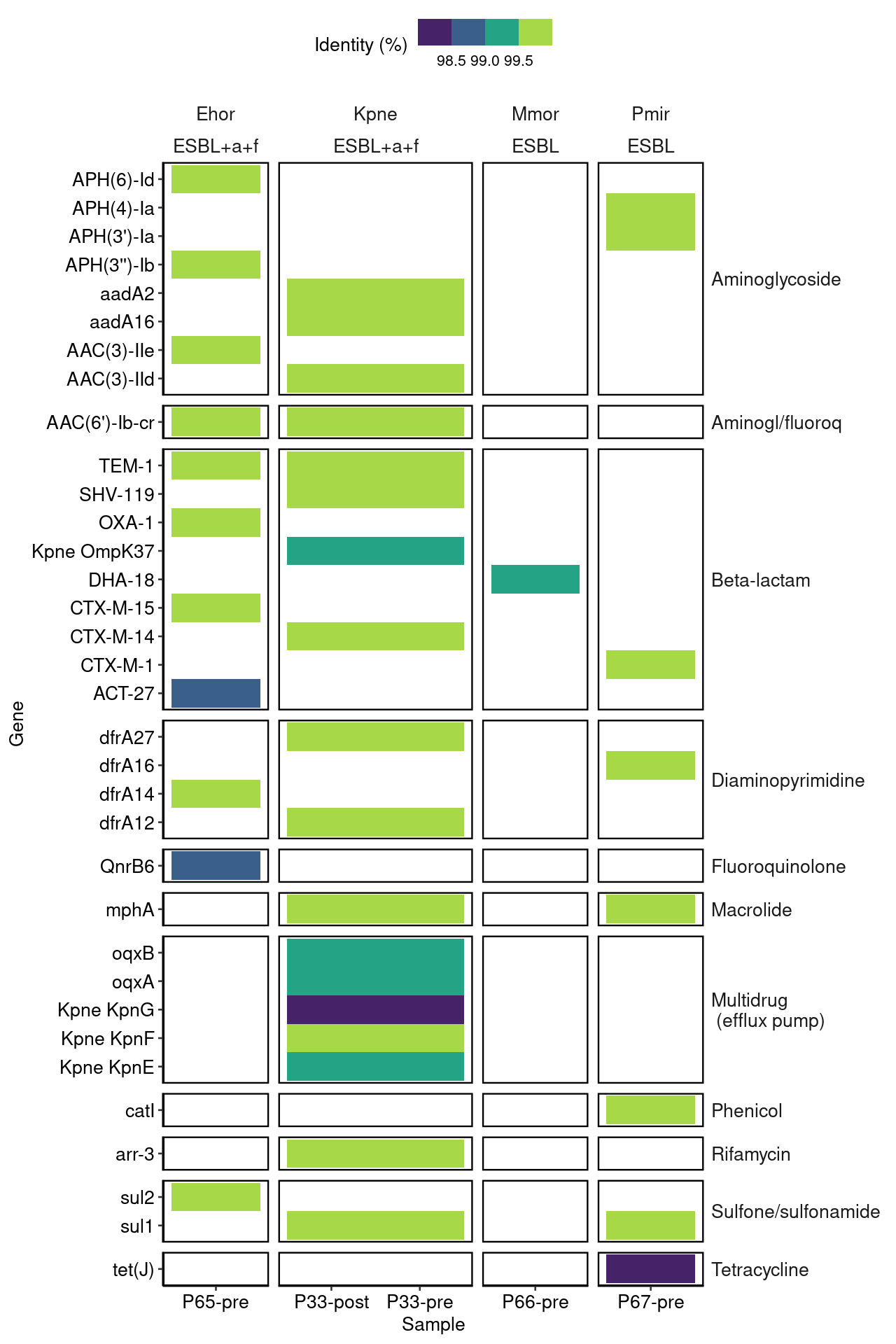


**Fig. S3. Detected antibiotic resistance genes in extended-spectrum beta-lactamase producing *Enterobacter hormaechei_A*, *Klebsielle pneumoniae*, *Morganella morganii* and *Proteus mirabilis* isolates.** One *E. hormaechei_A* strain was isolated from a patient pre-FMT, one *K. pneumoniae* was isolated from the same patient pre- and post-FMT, one *M. morganii* from one pre-FMT, and one *P. mirabilis* from one pre-FMT. Summarised by antibiotic class, colours represent nucleotide BLAST identity.
Ehor: *Enterobacter hormaechei_A*, Kpne: *Klebsiella pneumoniae*, Mmor: *Morganella margannii*, Pmir: *Proteus mirabilis*, ESBL: extended-spectrum beta-lactamase-producing, ESBL + a+f: ESBL-producing and aminoglycoside and fluoroquinolone resistant, pre: sample collected the day before FMT, post: sample collected ~3 weeks after FMT

**
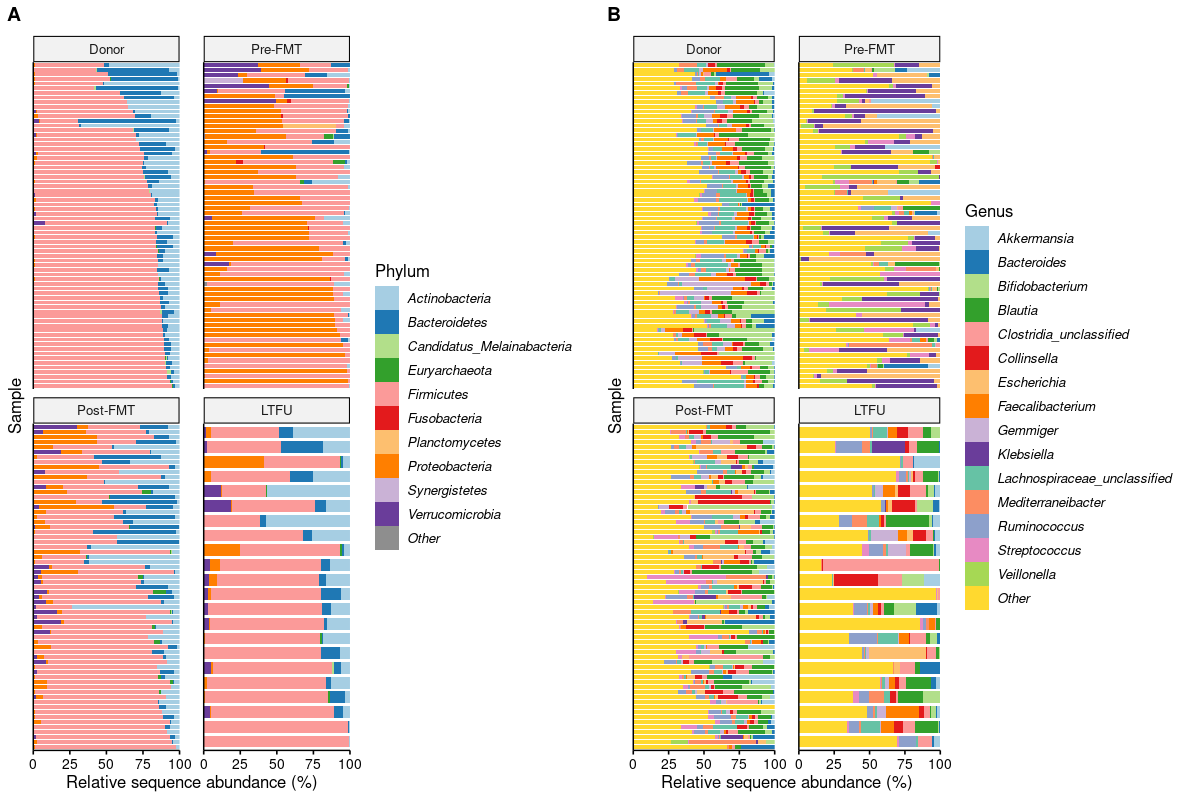
**

**Fig. S4. Taxonomic composition of faecal metagenomes of FMT donors and recipients.** Stool samples of healthy donors and FMT recipients were subjected to metagenomic sequencing and taxonomic profiles determined using MetaPhlAn4. The figure shows resulting composition at the A) phylum-rank and B) genus-rank.


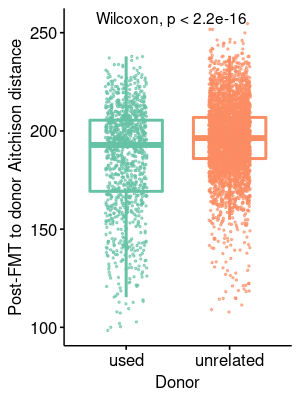


**Fig. S5. Aitchison distance from 63 post-FMT recipients’ species composition to 8 used and unrelated donors.** To determine if the microbiota shift in FMT recipients is at least partly attributable to the FMT or the effect of antibiotic cessation, we compared the recipients’ microbiota composition to the composition of the donor that was used for their FMT and other, unrelated donors.


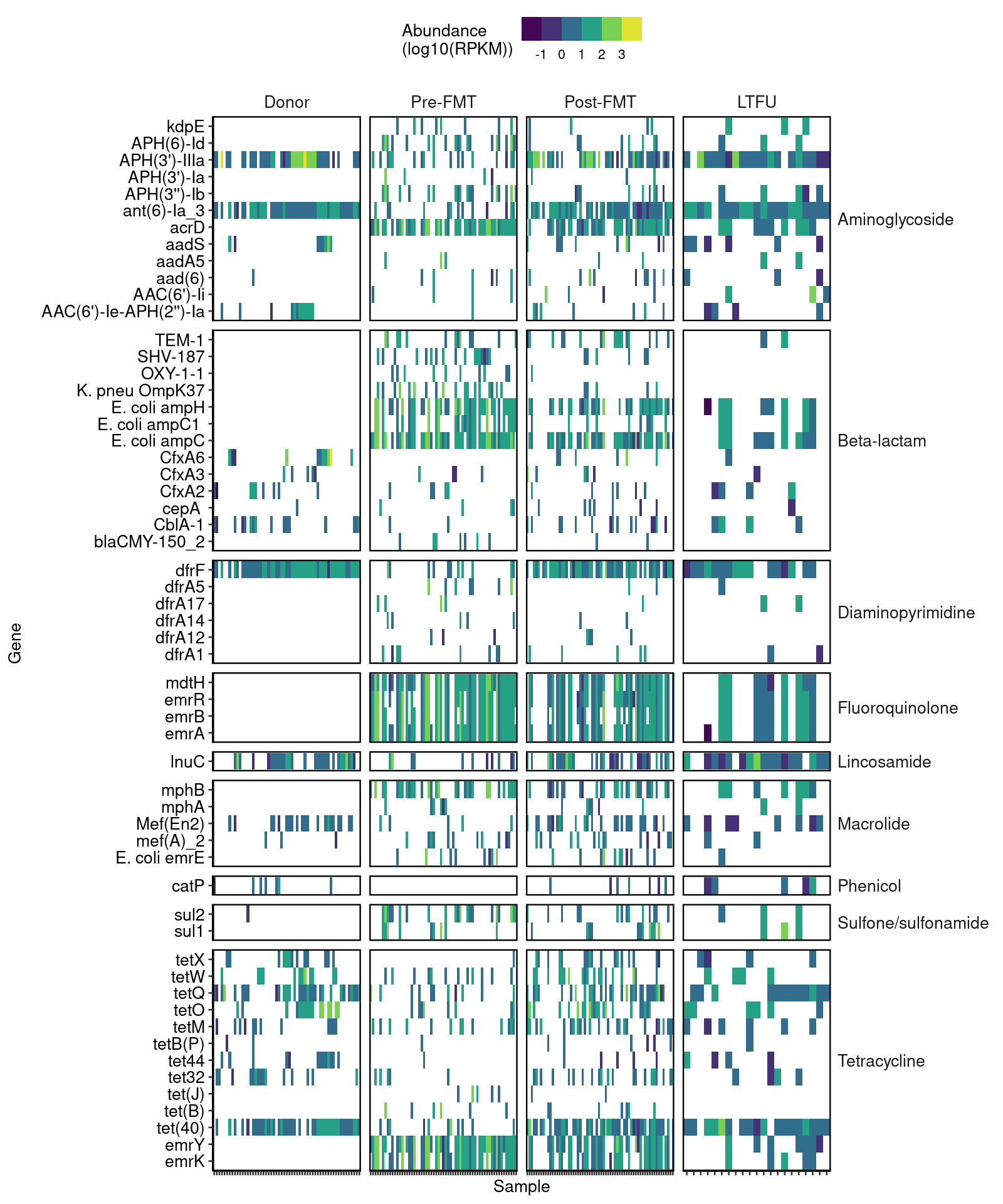


**Fig. S6. Occurrence and abundance of antibiotic resistance genes in faecal metagenomes of FMT donors and recipients.** Faecal metagenomes were screened for the presence of antibiotic resistance genes using the same approach as the whole-genome sequencing isolates (ABRicate with CARD and ResFinder). The heatmap shows abundance (blue-yellow) or absence (white) of antibiotic resistance genes from selected antibiotics classes in metagenomes of donors, or recipients one day before or three weeks after FMT, or at the long-term follow-up at roughly 1-3 years after FMT. For the clarity of the figure, only genes are shown that were detected at least 7 times.


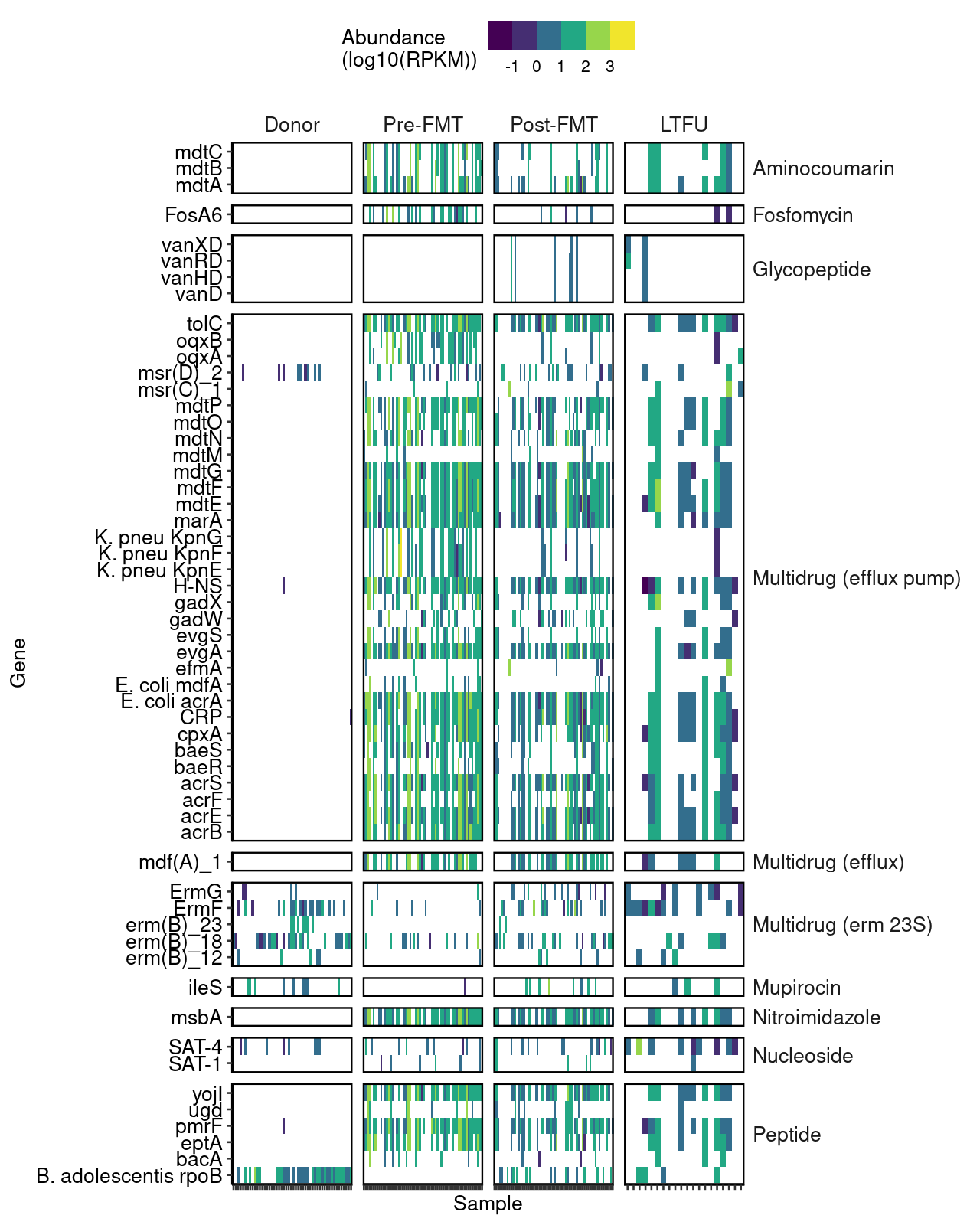


**Fig. S7. Occurrence and abundance of antibiotic resistance genes of other classes in faecal metagenomes of FMT donors and recipients.** Only genes are shown that were detected at least 7 times.


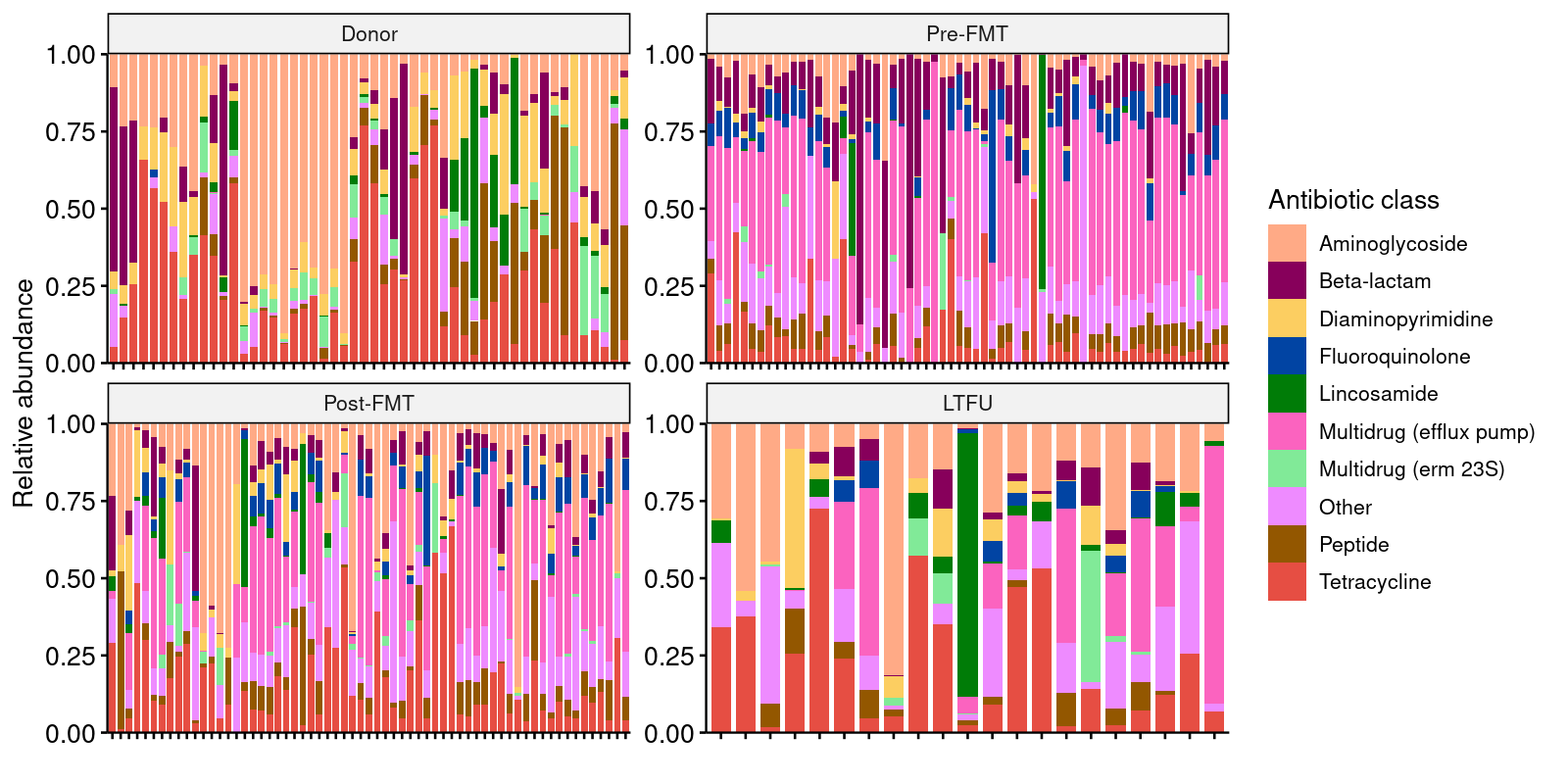


**Fig. S8. Resistome composition as relative abundance of antibiotics classes.** To gain more insight in the composition of the resistome, we visualised relative abundances of antibiotic resistance genes grouped by antibiotic classes.


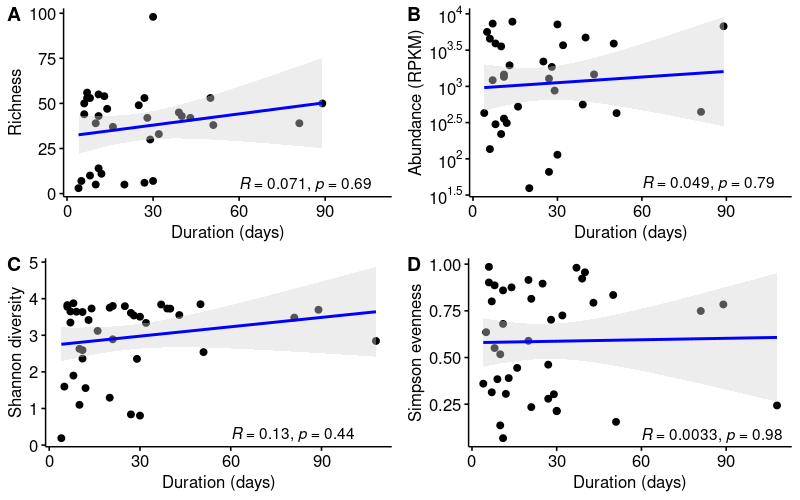


**Fig. S9. Resistome parameters compared with duration of vancomycin pre-treatment in days.** We examined if duration of vancomycin had an effect on resistome parameters by visualising for each patient (n=52) treated with vancomycin the duration of the treatment against resistome richness (A), total relative abundance (B), Shannon diversity (C) and Simpson evenness (D). Then we calculated Spearman correlations to calculate correlations. Blue lines indicate linear regression model, grey areas are 95% confidence intervals.


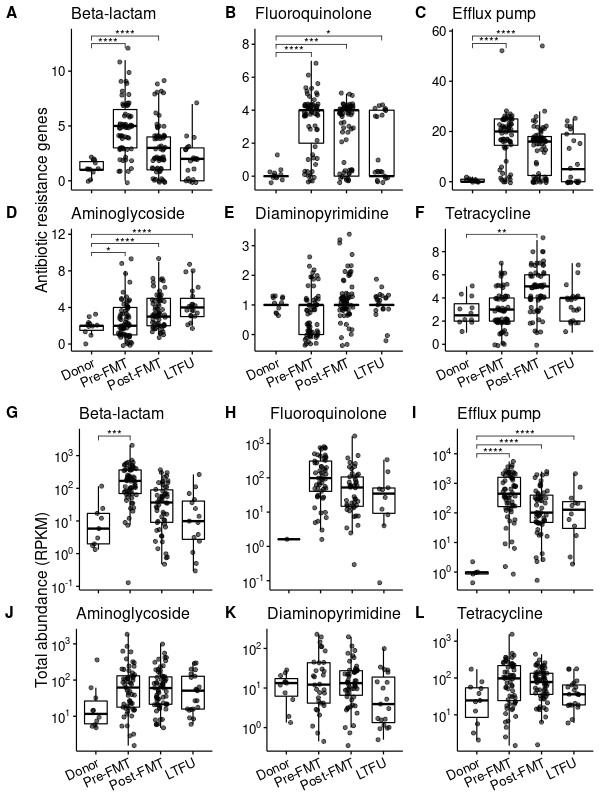


**Fig. S10. Richness and abundance of antibiotic genes of selected classes.** To illustrate differences in dynamics between antibiotic resistance genes of different classes, we highlight richness (A-F) and total abundance (G-L) of six selected classes. The first three are common and abundant in patients, and show a decrease in abundance after FMT. The other three are common in donors and are likely transferred to patients, which is indicated by an increase in richness and no decrease in abundance after FMT. Statistically significant differences are indicates by asterisks, *: p < 0.05; **: p < 0.01; ***: p < 0.001; ****: p < 0.0001.


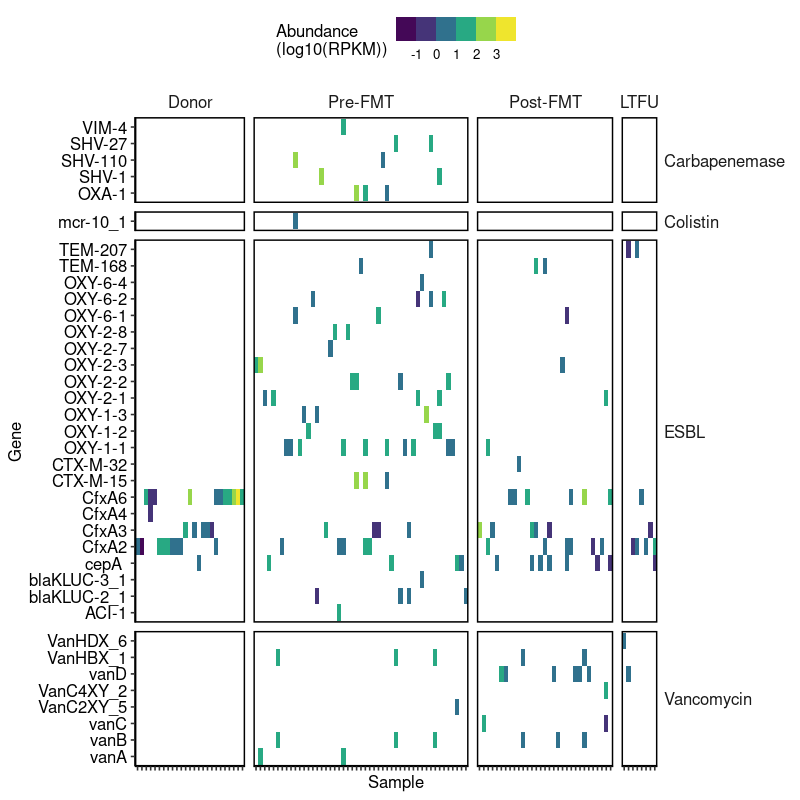


**Fig. S11. Antibiotic resistance genes of high clinical importance.** We screened metagenomes of FMT donors and recipients for the presence of antibiotic resistance genes that require special attention when present in a pathogen. This heatmap lists all the genes we detected that fit that category.

**
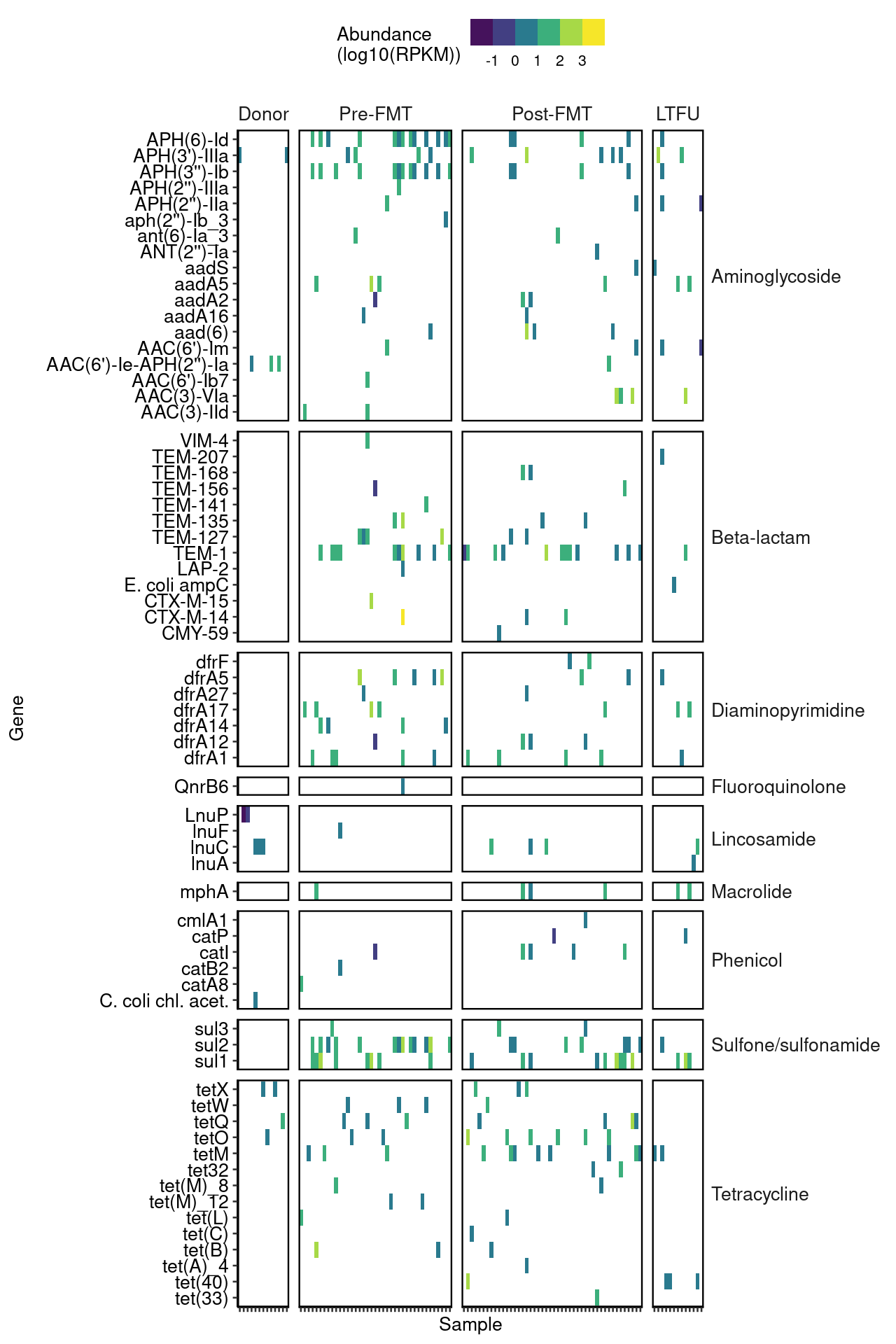
**

**Fig. S12. Overview of antibiotic resistance genes predicted to be on plasmids (part 1/2).** From all antibiotic resistance genes we detected in metagenomes, this figure shows genes that are predicted to derive from plasmids from the same antibiotic classes as Fig. S6. The figure shows n=13 donor samples from 7 different donors, n=39 pre-FMT patient samples, n=45 post-FMT samples and n=13 LTFU samples.

**
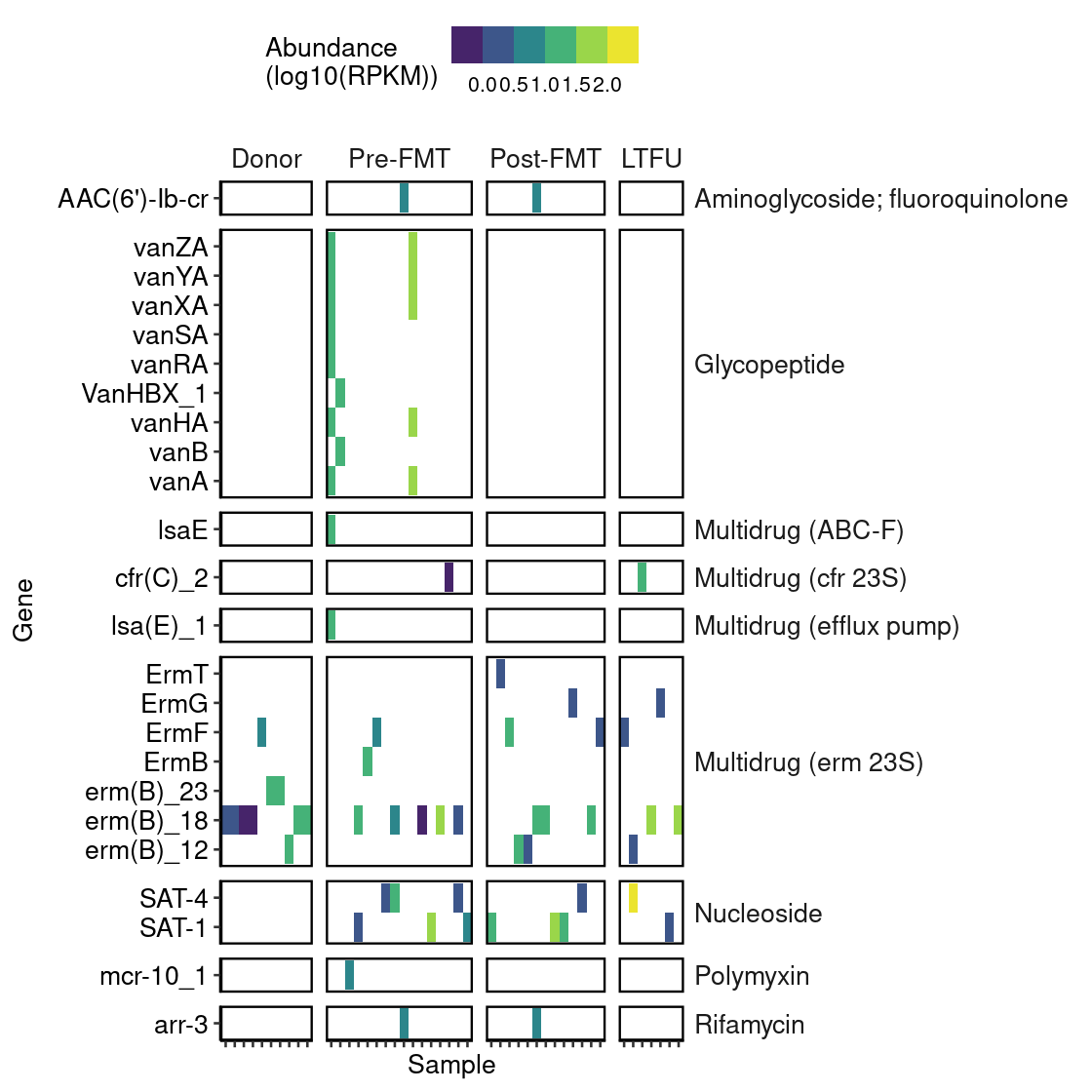
**

**Fig. S13. Overview of antibiotic resistance genes predicted to be on plasmids (part 2/2).** From all antibiotic resistance genes we detected in metagenomes, this figure shows genes that are predicted to derive from plasmids from the same antibiotic classes as Fig. S7. The figure shows n=10 donor samples from 4 different donors, n=16 pre-FMT patient samples, n=13 post-FMT samples and n=7 LTFU samples.
