# Supplementary material for: Long-term beneficial effect of faecal microbiota transplantation on colonisation of multidrug-resistant bacteria and resistome abundance in patients with recurrent Clostridioides difficile infection
Source: Genome Med. 2024 Feb 28;16:37. doi: 10.1186/s13073-024-01306-7 (PMC10902993; doi:10.1186/s13073-024-01306-7)
Supplement: Supplementary file 2 — Additional file 2: Supplementary results. Additional information regarding the detection of antibiotic resistance genes encoding carbapenemases, ESBL, and colistin and vancomycin resistance. [file 13073_2024_1306_MOESM2_ESM.docx]

# Supplementary results

*Potentially hazardous antibiotic resistances*

Certain antibiotic resistance classes are particularly hazardous should they be present in pathogens because they may lead to difficult to treat infections. These include carbapenemases, ESBL, vancomycin and colistin. Since we cannot determine the species of origin of all resistance genes we have found in metagenomes, we here make note of all resistance genes of these classes.

We detected one carbapenemase gene (*VIM-4*) in an rCDI patient before FMT (supplementary figure 11). The *VIM-4* gene was located on a 4.8kb contig, predicted to be a plasmid (supplementary figure 12). This gene was not found after FMT.

As for ESBL genes, we detected 26 *CfxA* (2, 3, 4 and 6) genes in donors as well as one *cepA* gene (supplementary figure 11). All these ESBL genes may be naturally present in *Bacteroides fragilis*, *B. caccae* or *Prevotella buccae* and are not thought to be of direct clinical importance (http://www.bldb.eu [1]). Within the patient group, we found *CTX-M-15, CTX-M-32*, *OXA-1*, and 50 *OXY, SHV* and *TEM* genes. Of these, only the *CTX-M-15* and *TEM-168* genes were predicted to be plasmid-derived (contigs were approximately 5kb and 10kb long, respectively; supplementary figure 13). Before FMT we detected a total of 69 ESBL genes in patients and 31 after FMT. Of these, we find only *OXY­* (four times, on chromosomes of Gammaproteobacteria) genes and *TEM-168* (twice, on plasmids) in post-FMT patient faecal metagenomes. Of the 136 ESBL genes detected in total, only 25 were found on contigs that could be classified to the genus rank. These genera include *Bacteroides*, *Kluyvera*, *Prevotella* and *Veillonella*. None of these matches (aerobically) cultured multidrug-resistant bacteria (Table 1).

Genes conferring resistance to vancomycin were never detected in donors, while 7 patients were found to have one or more *van* genes (supplementary figure 11). *VanA* and *vanB* were found in 2 and 3 rCDI patients before FMT, respectively, while *vanB* and *vanC* were detected in 3 and 2 patients after FMT. *VanA* was not detected in patients’ faeces after FMT. The 3 *vanA* genes were located on 7.5, 18 and 26 kb long contigs predicted to be plasmids, each of which shared homology with a plasmid identified in *Enterococcus faecium* (supplementary figure 13). This may indicate presence of a vancomycin resistant *Enterococcus* that was not detected by culture. Contrastingly, our culturing detected a vancomycin resistant *Enterococcus faecalis* with *vanA* gene cluster (*van[HRSXYZ]A*) in a post-FMT patient stool, of which we could not detect genes in the metagenome. The remaining vancomycin resistance genes were most probably of chromosomal origin, of which one *vanC*-containing contig was classified as belonging to *Enterococcus_D gallinarum*, and two *vanB* genes to *Collinsella*.

Finally, one gene conferring colistin resistance was found in the metagenome of an rCDI patient before FMT (supplementary figure 11). This is the gene *mcr-10_1­*, which derived from a 32kb plasmid (supplementary figure 13). We cannot determine the species that carried it, although we found it shares homology with a sequence previously found in *Enterobacter cloacae*. We could not detect *mcr-10_1* after FMT, so it may have disappeared from the patient.

# Supplementary references

1. Naas T, Oueslati S, Bonnin RA, Dabos ML, Zavala A, Dortet L, Retailleau P, Iorga BI: **Beta-lactamase database (BLDB) - structure and function**. *J Enzyme Inhib Med Chem* 2017, **32**(1):917-919.
